# On latches in biological systems: a comparative morphological and functional study of the retinaculum and the dens lock in Collembola

**DOI:** 10.1186/s12983-023-00491-2

**Published:** 2023-05-09

**Authors:** Birk Rillich, Fábio G. L. Oliveira

**Affiliations:** Institut Für Biowissenschaften, Universität Rostock, Allgemeine Und Spezielle Zoologie, Universitätsplatz 2, 18055 Rostock, Germany

**Keywords:** Biomaterials, Biomechanics, Resilin, Spring mechanism, Springtails

## Abstract

**Background:**

Springtails have the ability to jump using morphological structures consisting of a catapult, the furca, and a latching system constructed with interaction of the retinaculum and the dens lock. The retinaculum engages in the furca at the dens lock in order to form a spring mechanism. They exhibit diversified morphological traits that serve as adaptations to a variety of terrestrial strata and aquatic surface environments. This comparative morphofunctional study centered on the retinaculum and the furcular region of the dens lock aims to describe the morphological variation between taxa and provide insights into the functional dynamics of the latching mechanism at work in the jumping apparatus. Using SEM, µCT and cLSM, we compared representatives of Collembola taxa, Poduromorpha (*Neanura muscorum* and *Podura aquatica*), Symphypleona (*Dicyrtomina ornata*) and Neelipleona (*Megalothorax minimus*), and examined extracts of the environment in which they were collected.

**Results:**

A retinaculum is absent in *N. muscorum*, although vestigial muscles were found. Abdominal musculature varies significantly, being more abundant in springtails with clear segmentation (*N. muscorum* and *P. aquatica*), and reduced in springtails with fused segmentation (*D. ornata* and *M. minimus*). The M.a-ret varies as regards architecture and point of connection with the ramus, which is lateral in *P. aquatica* and median in the other species studied. The number of teeth in the retinaculum ramus also varies between three in *M. minimus* and four in the other species. The dens lock of all species studied has two locks and two furrows.

**Conclusions:**

The retinaculum and dens lock interact in a key-lock relationship. The latching and unlatching mechanism from the retinaculum and dens lock appear to be similar in all the taxa examined, occurring by muscle force. This leads us to question the hypothesis that hemolymph pressure may be a force generator in jumping. We offer a reconstruction of the ground pattern of the retinaculum and dens lock and, in addition, an explanation of their functioning and the interaction between them. Finally, we frame the interaction between the retinaculum and the dens lock as a latch in a biological system, a mechanism which functions by force of physical contact.

**Supplementary Information:**

The online version contains supplementary material available at 10.1186/s12983-023-00491-2.

## Background

Springtails are among the most numerous and widely distributed arthropods in the world [5]. About 9300 different species of these small wingless hexapods (0.12–17 mm) have been described so far, occurring in different habitats on all continents, including Antarctica [4, 5, 21, 49]. Due to their tiny size, they serve as food for various animals, including mites, pseudoscorpions, spiders and beetles [39, 41, 47, 51]. Escape from these predators is considered the main reason why the jumping apparatus developed in springtails [13, 23, 29].

Due to their vertical distribution profile, springtails can be classified as: a) euedaphic, living in the soil layer (body with trunk and reduced appendages); b) hemi/epiedaphic, living in the soil layer amongst leaf litter residues (regular body size); c) epigeic: living in plants/trees or even in aquatic environments (body with well-developed trunk and appendages) [41]. A variety of body shapes are found among springtails depending on the environment in which they live, and some studies have shown that jumping biomechanics also vary between representatives of the different habitats in terms of speed, force, height and angle [13, 33, 45, 46].

The spring mechanism in springtails includes a latching system in which the retinaculum is capable of actively holding the components of the spring (including the basal plates and sclerites) when it itself is latched on the dens lock, a region of the furca [32]. Both structures originate on the ventral surface of the abdomen, the retinaculum on the third and the furca on the fourth segment [5, 16, 17, 29, 32, 41]. The retinaculum has been described as an abdominal structure with an unpaired proximal part known as the corpus tenaculi and a paired distal part known as the ramus, in which several teeth are present [5, 20, 29, 32]. Although developments have been made into understanding the functioning of the retinaculum in Entomobryomorpha springtails [29, 32], little is known about its architecture and functioning in representatives of other groups. According to Manton [29], the contraction of intersegmental longitudinal muscles could cause the segments to overlap, creating a hemolymphatic pump which could then act to (1) distend the retinaculum, and (2) confer force at the moment of the jump. However, no morphological evidence was provided to support this hypothesis. Eisenbeis & Ulmer [17] performed ectomy experiments (cutting off parts of the furca, legs, thorax and abdomen) to test the role of hemolymphatic pressure in jumping efficiency, concluding that the animal could still jump, but with some loss of efficiency. Oliveira [32], after carrying out a detailed comparative morphological study of the jumping apparatus of *Orchesella cincta* (Linnaeus, 1758)  [26] in states of extended or flexed furca, rejected the hypothesis that hemolymphatic pressure acts on the functioning of the retinaculum since no evidence in support of it was found. He concluded that the distention of the retinaculum is made possible by muscle force alone. However, it was noted that the segments are more spaced and the muscles relaxed in the flexed furca phase, whereas the muscles are contracted and the segments overlap when the furca is extended, thus reinforcing Manton's hypothesis [29] about the potential presence of a hydrostatic pump as a mechanism working in the jump. Nothing is known so far about the functioning of the retinaculum in groups of springtails other than *O. cincta* [32] and *Tomocerus* [29]. When it comes to segmented springtail orders (such as Poduromorpha) and those with fused segmentation (Symphypleona and Neelipleona), not only is the functioning of the retinaculum and dens lock a mystery, the architecture and composition of the muscular system also remain to be investigated.

**Fig. 1:**
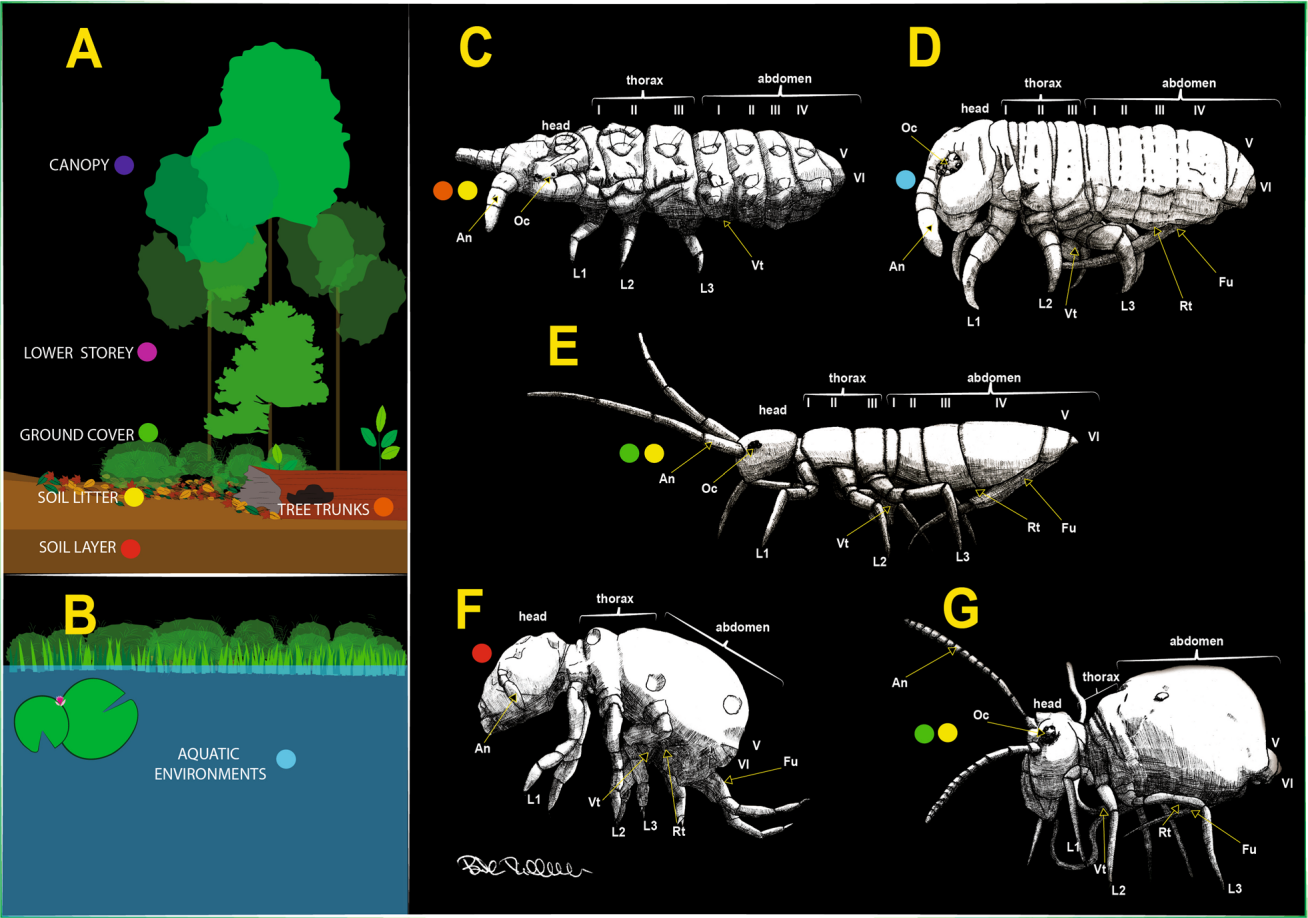
Scientific illustration showing vertical and horizontal distribution of springtails in landscapes, and general body morphology across the different taxa. **A** Terrestrial vertical distribution and **B** Aquatic environments; **C** *Neanura muscorum* (Templeton, 1835) [48], **D** *Podura aquatica* Linnaeus, 1758 [26], **E** *Orchesella cincta* (Linnaeus, 1758) [26], **F** *Megalothorax minimus* Willem, 1900 [50], **G** *Dicyrtomina ornata* (Nicolet, 1842). An: antenna; Oc: compound eyes; L: legs; Vt: ventral tube; Rt: retinaculum; Fu: Furca; Symbols, red: soil layer; orange: tree trunks; yellow: soil litter; green: ground cover; pink: lower storey; purple: canopy; blue: aquatic environments

When Longo et al. [27] asked how latches mediate the dynamics of energy release, one of their conclusions was that the duration and kinematic profile of latch release are influenced by the shape of the latch and the type of physical force present in it. This can vary within biological systems, for example from contact forces in the Mantis shrimp [36] to geometric forces in frogs [1]. Following Ilton et al. [24] and Longo et al. [27]’s reflections on how a latch controls the dynamics of energy release in a spring, we ask the following questions: (1) How does the shape of the retinaculum and dens lock differ between springtails living in different strata of the environment? (2) What is the retinaculum and its musculature like in species in which a furca is absent? (3) Do the retinaculum and dens lock function independently? (4) What structures of the retinaculum interact with the dens lock at the moment of latching? (5) Despite the morphological variation present, would it be possible to define a ground pattern for the retinaculum and the dens lock? (6) Could the mechanism of attaching or releasing the furca to the retinaculum be framed as a latch mechanism in biological systems? It is expected that (1) the shape of the retinaculum and dens lock will vary according to the habitat in which the species lives, but the latch mechanism will be similar across habitats; (2) the retinaculum musculature will be vestigial in species in which the furca is absent; (3) although retinaculum and dens lock are independent structures, the latching mechanism only works with the interaction of these two; (4) the retinaculum has structures at the ramus that fit the dens lock as in a key-lock relationship; (5) despite the morphological variation present, it is possible to define a ground pattern for the retinaculum and the dens lock; (6) it is possible to understand and describe the main latching mechanism behind this retinaculum-dens lock relationship. To confirm or refute our expectations, we used scanning electron microscopy (SEM), confocal laser scanning microscopy (cLSM), and microtomography (µCT) in order to carry out 3D morphological reconstruction. The morphological regions of the jumping apparatus we focused on were the abdominal structures, the retinaculum, and the dens lock. We reconstructed, described, and tried to forge a comparative understanding of the functioning of internal and external tissues of the retinaculum and it is latching structure, the dens lock, in representatives of different taxa of springtails living in different environmental conditions (Fig. 1). The following species were chosen for examination: *Neanura muscorum* (Templeton, 1835) [48] (living on tree trunks), *Podura aquatica* Linnaeus, 1758 [26] (living on the aquatic surface), *Dicyrtomina ornata* (Nicolet, 1842) [52] (living on surface leaf litter) and *Megalothorax minimus* Willem, 1900 [50] (living below the ground surface).

## Results

### Outer morphology

#### *Neanura muscorum*

**Fig. 2:**
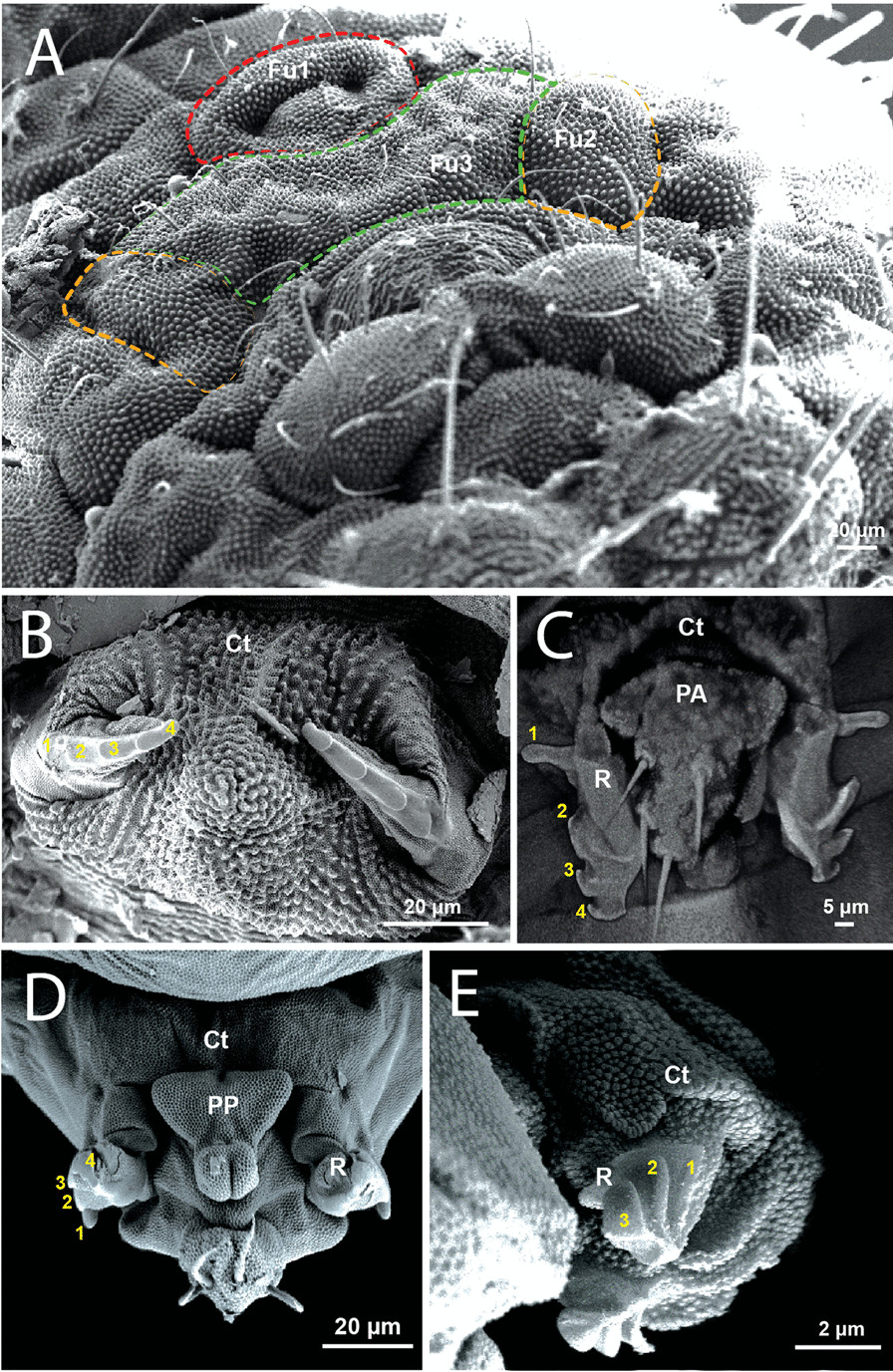
Comparative microscopy images of the retinaculum in Collembola (ventral view), **A** SEM image of *Neanura muscorum* (Templeton, 1835) [48], **B** SEM image of *Podura aquatica* Linnaeus, 1758 [26], **C** cLSM image of *Dicyrtomina ornata* (Nicolet, 1842) on 405 nm – anterior view **D** SEM image of *Dicyrtomina ornata* – posterior view, **E** SEM image of *Megalothorax minimus* Willem, 1900 [50]. Ct: corpus tenaculi,R: ramus; PA: anterior part; PP: posterior part. Dashed lines, red: Fu1; orange: Fu2; green: Fu3

*Neanura muscorum* is not able to jump and has a vestigial retinaculum and furca (Figs. 1C, 2A). Fu_1_ and Fu_3_ are unpaired parts of the vestigial furca. Fu_1_ can be described as an annular outward fold with an elevation in the center. Fu_3_ lies posterior to Fu_1_ and extends significantly further in the lateral direction than Fu_1_. Lateral to Fu_3_ is Fu_2_, which is separated from Fu_3_ by a longitudinal fold. Fu_2_ is roughly square in shape and is paired.

#### *Podura aquatica*

*Podura aquatica* has a well-developed retinaculum (Fig. 2B). The corpus tenaculi is significantly/visibly wider than it is long and is convexly curved on the anterior side while basically concave on the posterior side. It has an unpaired medial elevation that lies between the both rami. Small cuticular granules are concentric in elevation, with the center of the rings at their apex. Anterior and posterior to the elevation, the granules are arranged in longitudinal, parallel rows. Both rami are slightly bent medially and have four teeth laterally, the most proximal being the largest and the most apical the smallest. The teeth are roughly the shape of a right-angled triangle.

**Fig. 3:**
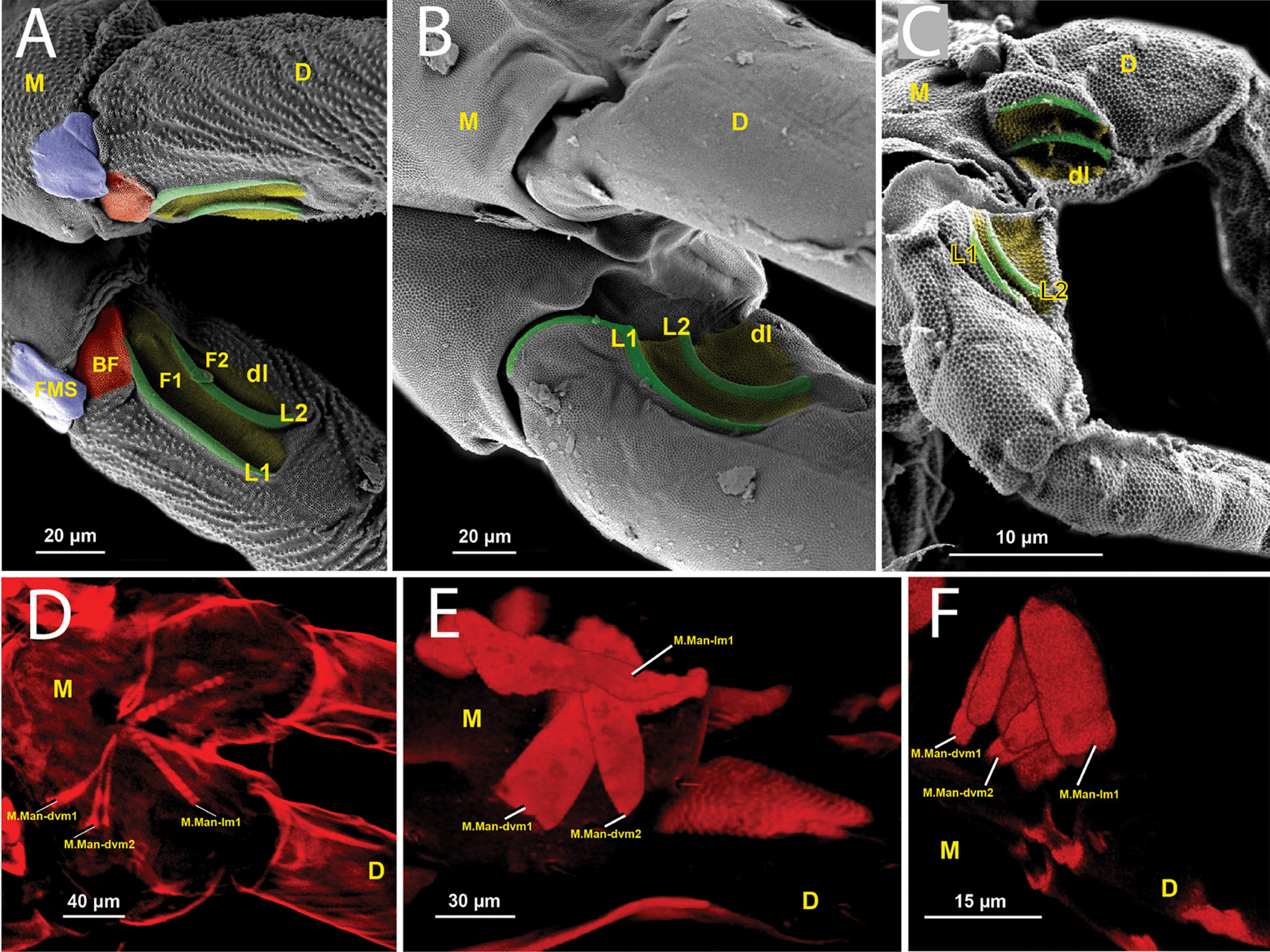
Microscopy images of the dens lock and the manubrial musculature in different taxa of Collembola. **A**–**C** SEM images of the dens lock (anterior view): **A** *Podura aquatica* Linnaeus, 1758 [26] **B** *Dicyrtomina ornata* (Nicolet, 1842) **C** *Megalothorax minimus* [50]. **D**–**F** cLSM images of the manubrial musculature on 555 nm: **D** *Podura aquatica* (posterior view); **E** *Dicyrtomina ornata* (lateral view); **F** *Megalothorax minimus* Willem, 1900 (lateral view). M: manubrium; D: dens; Dl: dens lock; L1: Lock 1; L2: Lock 2. Coloured structures, green: hooks L1 and L2; yellow: the furrows

When fully extended, the anterior side of the furca is located ventrally and the posterior side dorsally. The retinaculum attachment point, the dens lock, is shown in Figs. 3A and 11G, comprising locks and furrows. In *P. aquatica* there are two distinct longitudinal locks that rise steeply located anteromedially at the base of the dens. Posteriorly to each lock (L1 and L2) their respective furrows (F1 and F2) can be seen. The posterior lock (L2) is slightly curved along its entire length, bending convexly towards the medial sulcus. The anterior lock (L1), on the other hand, is only slightly bent anteriorly at its proximal end and then runs parallel to the dens distally. In the posterior region of the manubrium there is a manubrial furcular sclerite (FMS) which could potentially also be a lock since it has a furrow at the base of the dens, but the furrow is only a third of the length of F1 and F2. Here we propose the name basal furrow (BF) for this anterior region of the dens. Cuticular granules are distributed over the entire surface of the furrows, but are absent in the area of the retinaculum attachment point. The cuticle at the tip of the locks is smooth, the remainder bears the typical pattern present in the body of the retinaculum.

#### *Dicyrtomina ornata*

The retinaculum is divided into an anterior and a posterior part, with only the anterior part making contact with the sternite. The anterior part, seen from posterior, is approximately an upside down triangle, but with wing-like extensions that reach about three-quarters of the way down towards its apex (Fig. 2C). The posterior part is wide and triangular where it conforms to the anterior of the dens lock and narrows continuously distally until the lateral borders are parallel for a short distance (Fig. 2D). The rami are slightly bent outwards and have four teeth. The proximal tooth, also called the appendix [20], is externally oriented and finger-like in shape (Fig. 2C). The distal teeth together are oriented towards the sides and cover only about half of the lateral edges of the rami (Fig. 2D). The tips of the rami are slightly bent inwards (Fig. 2C, D).

The point of attachment of the retinaculum, the dens lock is shown in Figs. 3B and 11H. The dens lock has two grooves running from anteroproximal to posterodistal. The furrow located more posteriorly is the smallest, while the anterior one is twice its size. Steep locks separate the grooves. The locks are strongly convex to the anterior side. The upper edge of the locks is the only part of this structure where cuticular granules are not found.

#### *Megalothorax minimus*

The corpus of the retinaculum is shaped roughly like a posterior facing bisected cylinder with the side cut off. Anteriorly, the corpus extends into two lobes, which lie parallel to the sagittal plane (Fig. 2E). Posteriorly the lobes end parallel to the transversal plane and their most distal point is approximately above the center of the retinaculum. At the point of articulation between ramus and corpus there are lobe-like folds which nestle against the basal part of the ramus. The entire cuticle of the corpus exhibits the pattern of primary granules described in *N. muscorum.* The insertion point of the ramus is broad and the ramus narrow distally. There are three teeth on each ramus which are triangular plate-like in shape. One edge of the triangle is attached to the ramus obliquely, running from anterodistal to posteroproximal. The apex opposite this edge is directed anterolaterally and ventrally. The cuticle of the teeth is smooth and without the pattern of primary granules seen elsewhere on the cuticle of the corpus.

The point of attachment between the retinaculum and the furca, the dens lock consists of two furrows and two locks (Fig. 3C, 11I). The furrows run from anteroproximal to posterodistal. The posterior furrow is the smallest, but it is wider proximally than the other two furrows. The locks run in a slightly curvilinear shape, becoming convex toward the anterior. Only the upper edges of the locks do not show the typical pattern of cuticular granulation.

### Inner morphology

All the muscles labeled in the figures below and mentioned as abbreviations in the text are listed in Table 1. We reconstructed and described the muscles of the third abdominal segment (where the retinaculum occurs) and the musculature related to the dens lock, at the base of the furca. In globular taxa such as Symphypleona and Neelipleona with fused abdominal segmentation, it was not possible to attribute muscles precisely to the third segment. We also considered the intersegmental muscles with possible relation to the retinaculum, those which originate in or simply cross the third abdominal segment. Due to the complexity of handling specimens as small as *Megalothorax minimus*, it was not possible in the current study to examine both flexed and extended furca states in this taxon.

**Table 1:** Compilation of the muscles in vicinity of the retinaculum and their respective attachment points in the taxa studied

Name of the muscle	Origin	Insertion point
*Neanura muscorum (Templeton, 1835) *[48]		
M.IIa-vlm1	Ventromedial transition between Ia and IIa	Ventromedial muscle center IIIa
M.IIa-vlm2	Ventromedial transition between Ia and IIa	Ventromedial muscle center IIIa
M.IIa-vlm3	Ventromedial transition between Ia and IIa	Ventromedial muscle center IIIa
M.IIa-vlm4	Ventromedial transition between Ia and IIa	Ventromedial muscle center IIIa
M.IIa-isvlm1	Ventromedial transition between Ia and IIa	Ventromedial sternite IVa
M.IIa-isvlm2	Ventromedial transition between Ia and IIa	Ventromedial sternite IVa
M.IIIa-dlm1	Tergite dorsal in transition area between IIa and IIIa	Tergite dorsomedial in transition area between IIIa and IVa
M.IIIa-dlm2	Tergite dorsomedial in transition area between IIa and IIIa	Tergite dorsomedial in transition area between IIIa and IVa
M.IIIa-dvm1	Ventromedial sternite at base of retinaculum	Dorsomedial at tergite IIIa
M.IIIa-trm1	Ventromedial muscle center IIIa	Ventrolateral sternite IIIa
M.IIIa-trm2	Ventromedial muscle center IIIa	Ventrolateral sternite IIIa
M.IIIa-trm3	Ventromedial muscle center IIIa	Ventrolateral sternite IIIa
M.IIIa-trm4	Tergite dorsolateral in transition area between IIa and IIIa	Lateral between tergite and sternite IIIa
M.IIIa-ldvm1	Ventrolateral sternite IIIa	Dorsomedial at tergite IIIa
M.IIIa-ldvm2	Ventrolateral sternite IIIa	Dorsomedial at tergite IIIa
M.IIIa-ret	Ventrally at sternite IIIa	Ventromedial muscle center IIIa
M.IIIa-vlm1	Ventromedial muscle center IIIa	Ventromedial sternite transition between IIIa and IVa
M.IIIa-vlm2	Ventromedial muscle center IIIa	Ventromedial sternite transition between IIIa and IVa
M.IIIa-vlm3	Ventromedial muscle center IIIa	Ventromedial sternite transition between IIIa and IVa
*Podura aquatica Linnaeus, 1758 *[26]		
M.Ia-vlm1	Ventromedial transition between IIIt and Ia	Muscle center in transition area between IIa and IIIa
M.Ia-vlm2	Ventromedial transition between IIIt and Ia	Muscle center in transition area between IIa and IIIa
M.Ia-vlm3	Ventromedial transition between IIIt and Ia	Muscle center in transition area between IIa and IIIa
M.Ia-vlm4	Ventromedial transition between IIIt and Ia	Muscle center in transition area between IIa and IIIa
M.Ia-isvlm1	Ventromedial transition between IIIt and Ia	Ventromedial transition between IIIa and IVa
M.Ia-isvlm2	Ventromedial transition between IIIt and Ia	Ventromedial transition between IIIa and IVa
M.IIIa-ret	Lateral at inside of ramus	Ventromedial muscle center IIIa
M.IIIa-vlm1	Ventromedial muscle center IIIa	Ventromedial at sternite in transition area between IIIa and IVa
M.IIIa-vlm2	Ventromedial muscle center IIIa	Muscle center in transition area between IIIa and IVa
M.IIIa-dvm1	Ventromedial sternite at base of retinaculum	Dorsomedial at tergite IIIa
M.IIIa-dvm2	Ventrolateral at sternite IIIa	Dorsolateral at tergite IIIa
M.IIIa-dlm1	Tergite dorsal in transition area between IIa and IIIa	Tergite dorsomedial in transition area between IIIa and IVa
M.IIIa-dlm2	Tergite dorsomedial in transition area between IIa and IIIa	Tergite dorsomedial in transition area between IIIa and IVa
M.IIIa-isvlm1	Attached to M.III-dvm1 in transition area between IIa and IIIa	Tergite lateral in transition area between VIa and Va
M.IIIa-isvlm2	Attached to M.III-dvm1 in transition area between IIa and IIIa	Attached to M.IVa-trm1
M.IIIa-istrm1	Tergite dorsolateral in transition area between IIa and IIIa	Ventrolateral at sternite IIIa
M.IIIa-trm1	Ventromedial muscle center IIIa	Ventrolateral sternite IIIa
M.IVa-trm1	Attached to M.III-isvlm2	Ventrolateral at sternite at base of furca
M.Man-dvm1	Attached to anterior portion of the base of the manubrium	Posterior portion of manubrium
M.Man-dvm2	Attached to anterior portion of the base of the manubrium	Posterior portion of manubrium
M.Man-lm1	Attached to posterior portion of the base of the manubrium	Lateral anterior to dens
Dicyrtomina ornata (Nicolet, 1842)		
M.a-ret	Medial inside of ramus	Attached to M.a-rtrm1
M.a-rtrm1	Attached to M.a-ret	Muscle center in vicinity of retinaculum
M.a-rtrm2	Muscle center in vicinity of retinaculum	Ventrolateral at base of retinaculum
M.a-rtrm3	Anterior to retinaculum, at base of ventral tube	Muscle center in vicinity of retinaculum
M.a-rtrm4	Anterior to retinaculum, at base of the ventral tube	Muscle center in vicinity of retinaculum
M.a-rtrm5	Dorsolateral to retinaculum, at base of ventral tube	Muscle center in vicinity of retinaculum
M.a-rdvm1	Muscle center in vicinity of retinaculum	Dorsolateral at tergite
M.a-rdvm2	Muscle center in vicinity of retinaculum	Dorsal at tergite
M.a-rldvm1	Muscle center in vicinity of retinaculum	Dorsolateral at tergite
M.a-rldvm2	Muscle center in vicinity of retinaculum	Dorsolateral at tergite
M.a-rldvm3	Attached to M.a-rvlm2	Dorsolateral at tergite
M.a-rldvm4	Attached to M.a-rvlm2	Dorsolateral at tergite
M.a-rvlm1	Anterior to retinaculum, at base of ventral tube	Muscle center in vicinity of retinaculum
M.a-rvlm2	Muscle center in vicinity of retinaculum	Lateral at sternite
M.Man-dvm1	Medial to anterior most portion of base of manubrium	Lateral in central region of manubrium
M.Man-dvm2	Medial to central region of base of manubrium	Lateral in central region of manubrium
M.Man-lm1	Dorsal attached to anterior portion of base of manubrium	Lateral anterior to dens
*Megalothorax minimus Willem, 1900 *[50]		
M.a-dlm1	Dorsal anterior at abdomen	Dorsal in middle portion of abdomen
M.a-dlm2	Dorsal anterior at abdomen	Dorsal in middle portion of abdomen
M.a-dvm1	Attached to M.a-te	Dorsolateral at tergite
M.a-dvm2	Attached to M.a-te	Dorsolateral at tergite
M.a-te	Attached to M.a-ret	Attached to M.a-dvm1 and M.a-dvm2
M.a-ret	Medial inside of ramus	Attached to M.a-te
M.a-rtrm1	Attached to M.a-ret	Ventrolateral at sternite in vicinity of retinaculum
M.Man-dvm1	Dorsal to anterior portion of manubrium	Ventrolateral at base of manubrium
M.Man-dvm2	Dorsal to central portion of manubrium	Ventrolateral at base of manubrium
M.Man-lm1	Anterior at base of manubrium	Lateral anterior to dens

#### *Neanura muscorum*

**Fig. 4:**
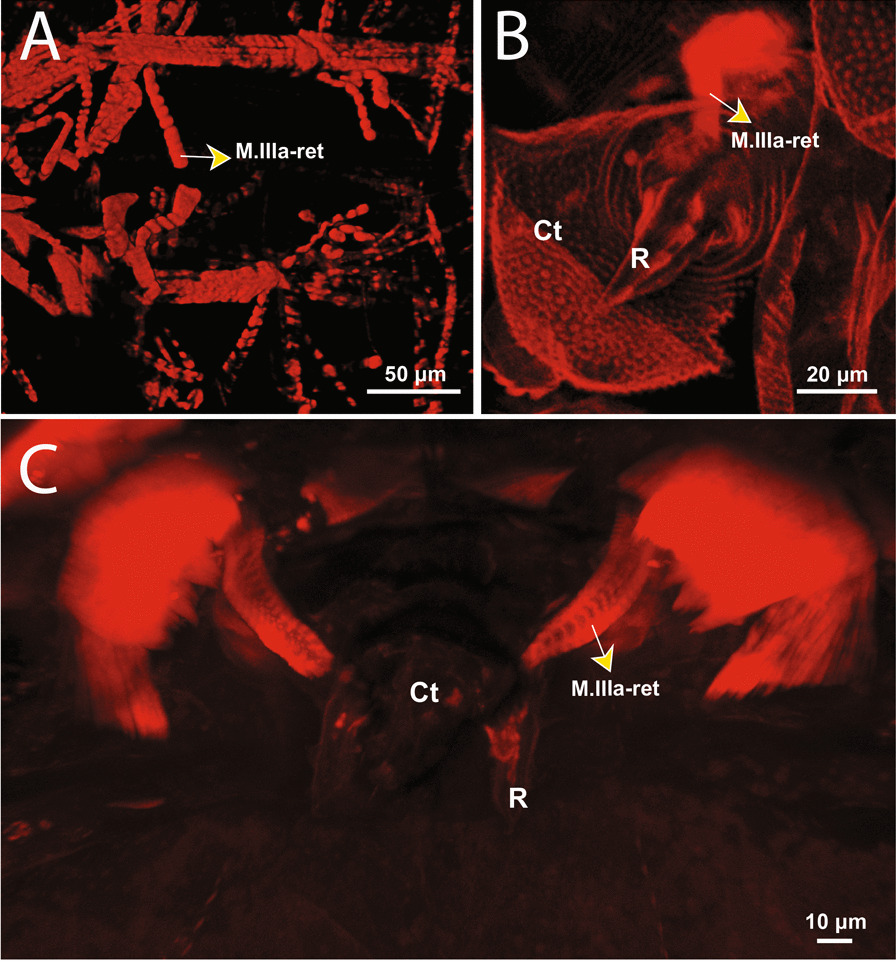
cLSM images of the retinacular muscle (M.III-ret), revealed by phalloidin staining at 555 nm (anterior ventral view). **A** *Neanura muscorum* (Templeton, 1835) [48], **B** *Podura aquatica* Linnaeus, 1758 [26], **C** *Dicyrtomina ornata* (Nicolet, 1842). Ct: corpus tenaculi; R: ramus

**Fig. 5:**
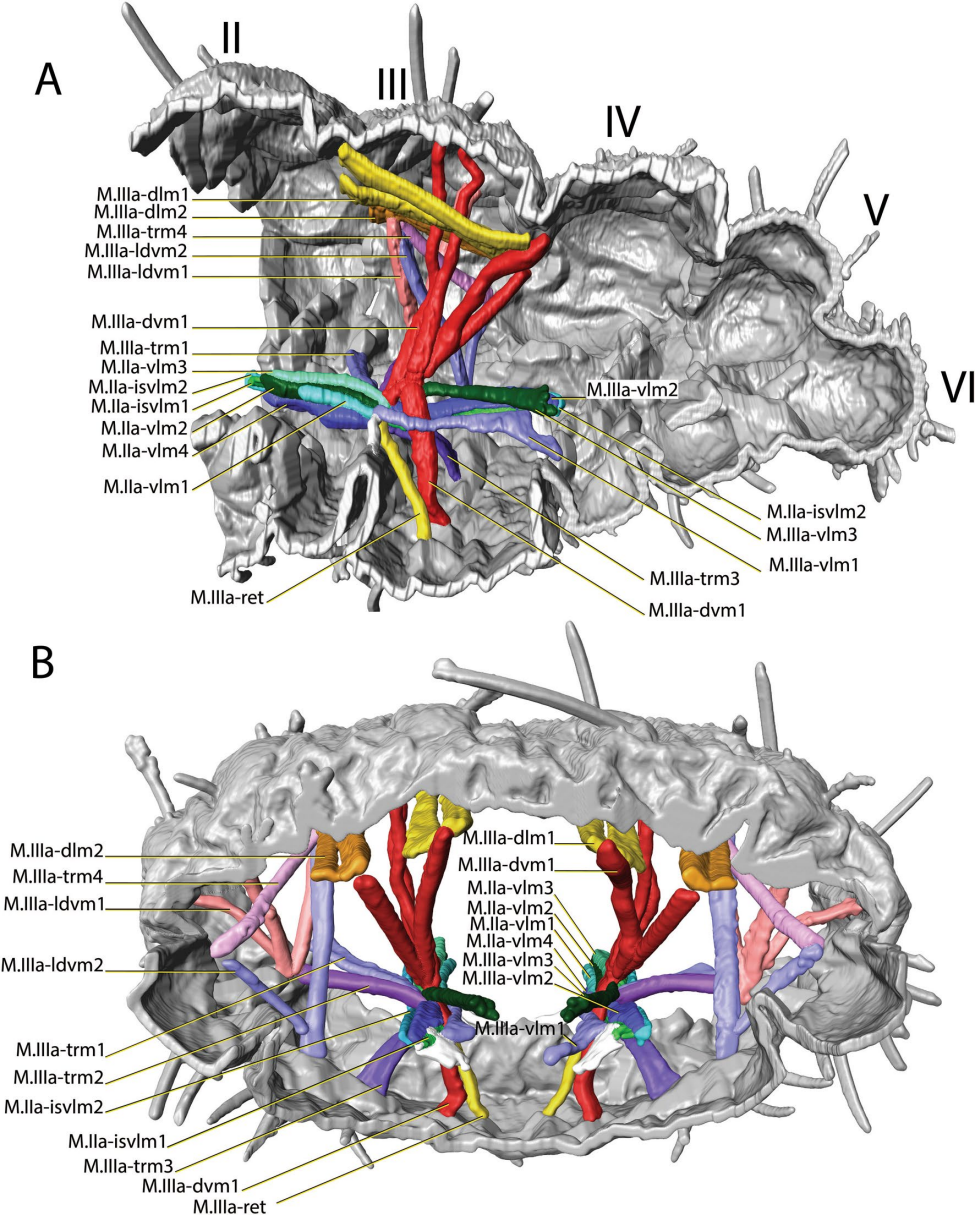
µCT-based morphological reconstruction of the abdominal segments, the jumping apparatus, cuticle and musculature of the retinaculum in *Neanura muscorum* (Templeton, 1835) [48]. **A** Cut-away longitudinal view, **B** Transverse view

The retinaculum muscle attaches ventrally to the inside of the anterior annular fold of Fu_1_ (Figs. 2A, 4A, 5A, B). Dorsally, it attaches to a muscle center which is located ventromedially in the transition area between IIa and IIIa. The retinaculum muscles are relatively long and similar in thickness to the rest of the muscles (Figs. 4A, 5A, B). The muscle system can generally be described as strongly interconnected. There are three longitudinal muscle bundle pairs, one located ventrally, one dorsally and one dorsolaterally. The muscle centers are strongly interconnected via transversal, longitudinal, dorsoventral muscles and they are always located in the transition area between the abdominal segments. It is noticeable upon examination of the cLSM images that muscles exhibit transverse striation (Fig. 4A).

#### *Podura aquatica*

**Fig. 6:**
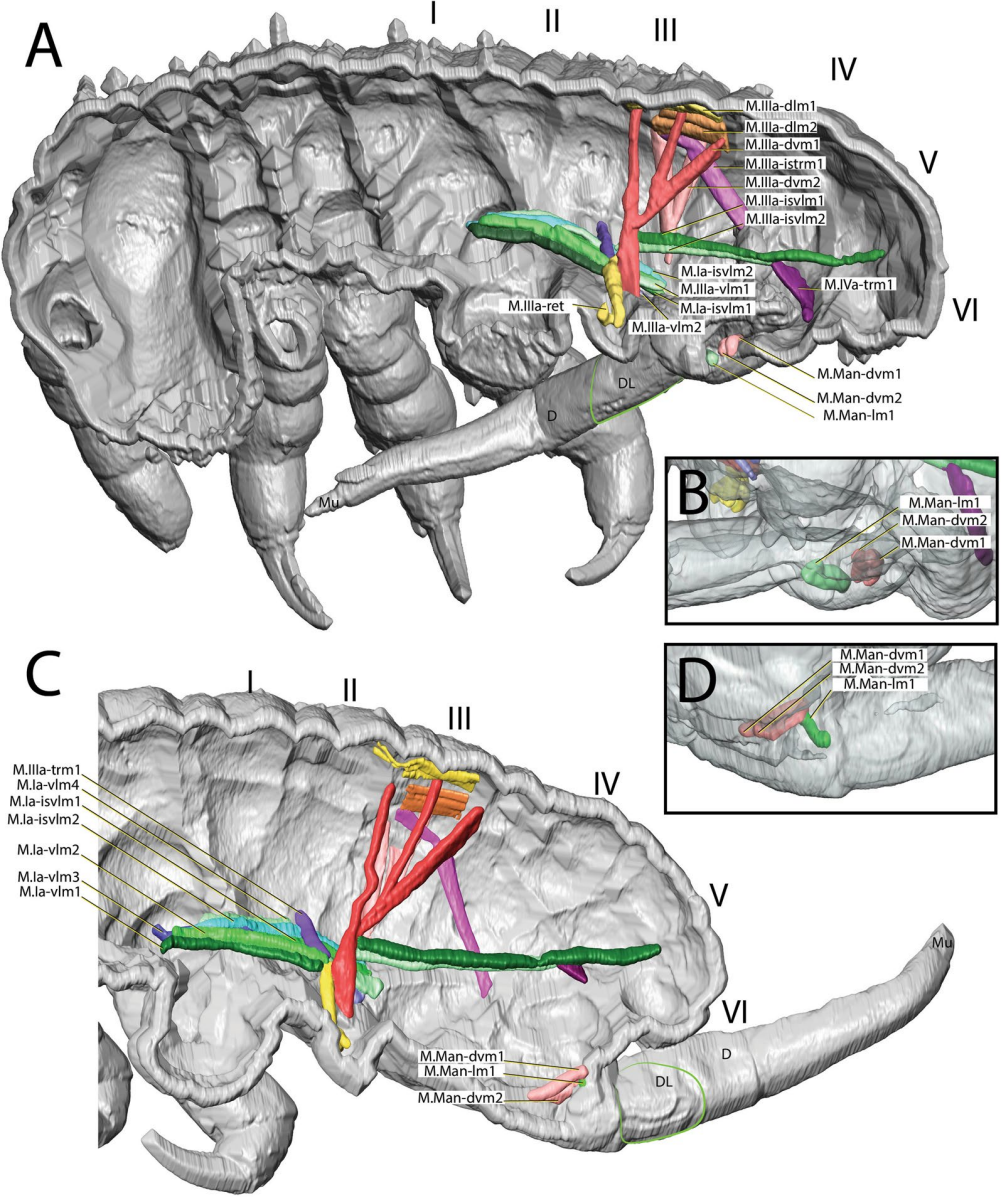
µCT-based morphological reconstruction of the abdominal segments, jumping apparatus, cuticle and retinacular musculature in *Podura aquatica* Linnaeus, 1758 [26]. **A** Cut-away longitudinal view—flexed furca, **B** External lateral view of the base of the furca and manubrial muscles—flexed furca; **C** Cut-away longitudinal view—extended furca; **D** External lateral view of the base of the furca and manubrial muscles—extended furca. D: dens; DL: dens lock; Mu: Mucro

**Fig. 7:**
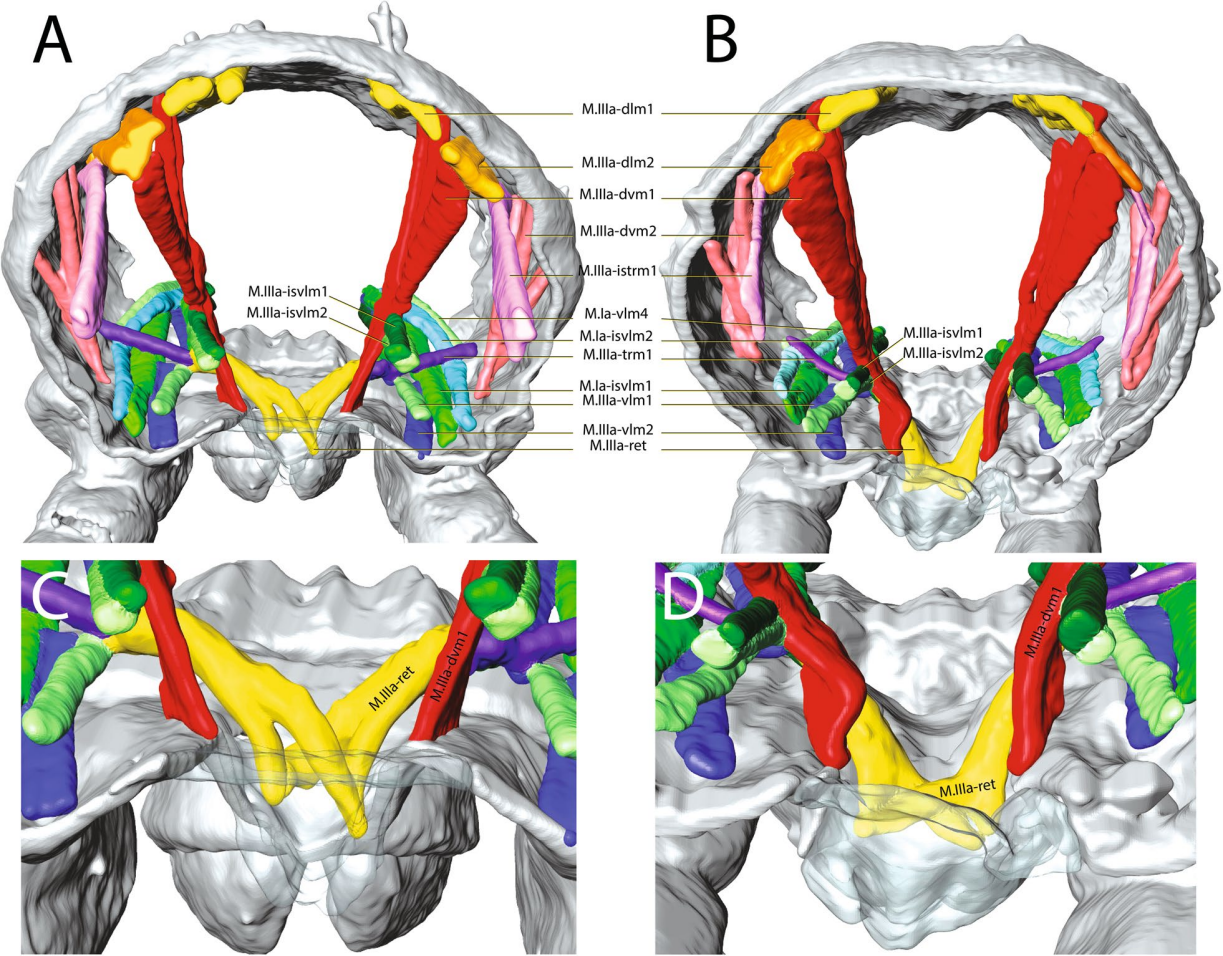
µCT-based morphological reconstruction of the abdominal segments, the jumping apparatus, cuticle and musculature of the retinaculum in *Podura aquatica* Linnaeus, 1758 [26]. **A** Transversal view – furca flexed, **B** Transverse view – furca extended; **C** Transverse view – furca flexed; **D** Transverse view – furca extended

M.IIIa-ret is bifurcated, dividing ventrally, while both sides are connected in the middle. Both muscle bundles insert laterally inside the branch (Figs. 4B, 6 and 7). Within the branch, one of the two branches attaches to the left half of the body and the other to the right half, causing a crossover.

The muscular system is similar in structure to that of *N. muscorum*. There are three pairs of longitudinal muscle bundles per segment, one located ventrally, one dorsally, and one dorsolaterally. However, the two dorsal muscle bundles are much closer to each other than in *N. muscorum*. In *P. aquatica*, the ventral longitudinal muscle bundles connect the muscle centers to each other. The dorsal muscle bundles, on the other hand, do not attach to the muscle centers but to the inner side of the cuticular folds that mark the borders between the tergites. The ventral longitudinal muscle bundles, unlike in *N. muscorum*, extend only to the end of the third abdominal segment. As observed in *N. muscorum*, the density of the dorsoventral musculature in the region of the fourth abdominal segment is significantly higher than in the other segments. These dorsoventral muscles are not noticeably thicker than the other muscles. *P. aquatica* is unique in having two ventral longitudinal muscles (M.IIIa-isvlm1 and M.IIIa-isvlm2) that run from the third abdominal segment posterior to the tergite at the transition between VIa and Va.

A comparison between the states furca flexed and furca extended showed that the two M.IIIa-ret of the rami are significantly thicker in the individual with the extended furca than in the individual with the flexed furca, which indicates that they are contracted. The branches of the two muscles touch medially when the M.IIIa-ret contracts. With the furca flexed, M.IIIa-dvm1 is visibly straighter at its ventral base and in its dorsal ramus than when the furca is extended (Figs. 6 and 7).

#### *Dicyrtomina ornata*

**Fig. 8:**
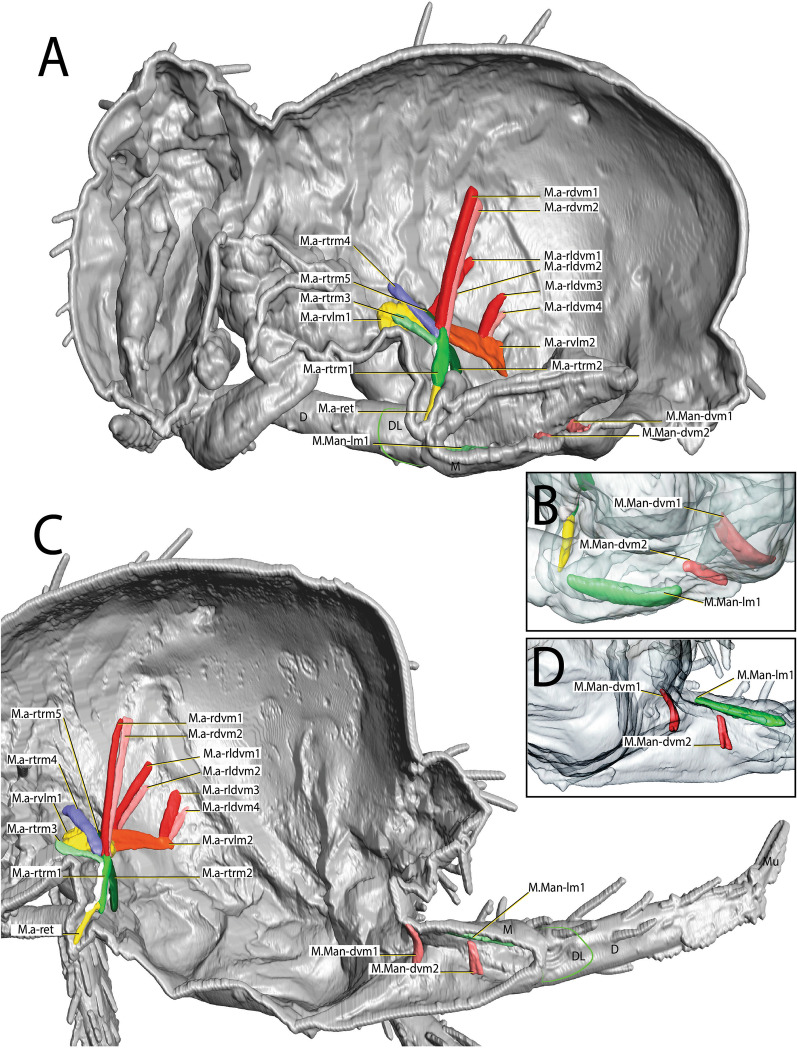
µCT-based morphological reconstruction of the abdominal segments, the jumping apparatus, cuticle and musculature of the retinaculum in *Dicyrtomina ornata* (Nicolet, 1842). **A** Cut-away longitudinal view—flexed furca; **B** External lateral view of the base of the furca and manubrial muscles—flexed furca; **C** Cut-away longitudinal view—extended furca; **D** External lateral view of the base of the furca and manubrial muscles—extended furca. M: manubrium; D: dens; DL: dens lock; Mu: Mucro

**Fig. 9:**
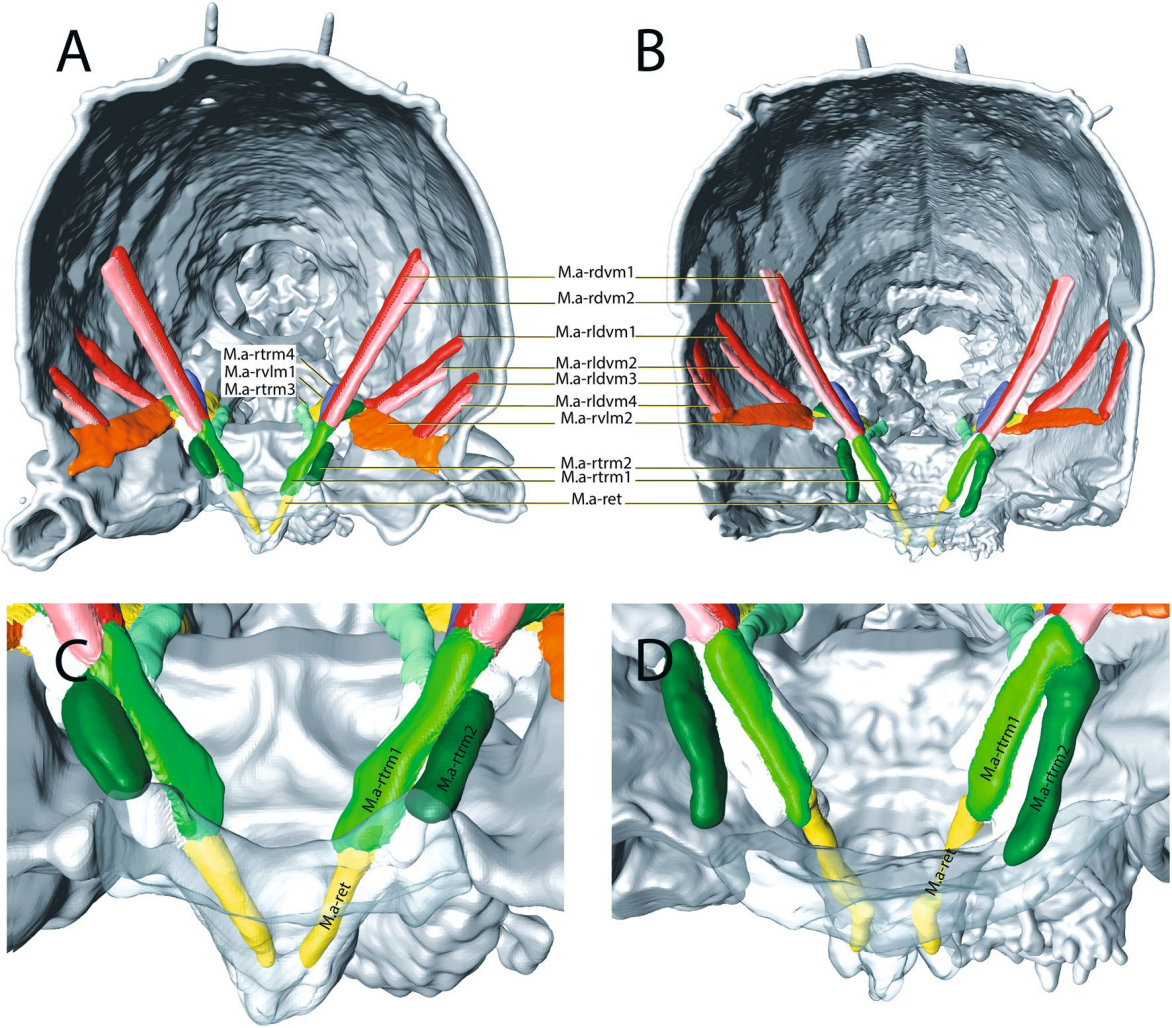
µCT-based morphological reconstruction of the abdominal segments, the jumping apparatus, cuticle and musculature of the retinaculum in *Dicyrtomina ornata* (Nicolet, 1842) – (Transversal view). **A** flexed furca; **B** extended furca; **C** flexed furca; **D** extended furca

M.a-ret attaches ventrally to the medial inner side of the ramus. Dorsally it is connected to M.a-rtrm1, which attaches to a muscle center (Figs. 4C, 8 and 9). As in *O. cincta*, the muscle of the retinaculum is not directly connected to the muscle center [32], the connection is mediated by transversal muscles (Figs. 8 and 9). Muscles M.a-ret and M.a-rtrm1 connect through a tendon that extends between the ends of the two muscles, acting as an anchoring structure and also separating them. The abdominal musculature in *D. ornata* is reduced compared to that in segmented taxa. The muscles that apparently work on the retinaculum extend from the anterior end of the abdomen to about a quarter of the way down the abdomen. In this area there are also many dorsoventral and transverse muscles which are similar in thickness to the rest of the muscles in this part of the abdomen.

A comparison of the states furca flexed and furca extended indicates that the muscle of the retinaculum is contracted when the furca is flexed and is visibly straighter than when the furca is extended. The same can be said of M.a-rtrm1, which runs in a straight line with the retinaculum muscle when the furca is flexed. When the furca is extended this is not the case (Figs. 8 and 9).

#### *Megalothorax minimus*

**Fig. 10:**
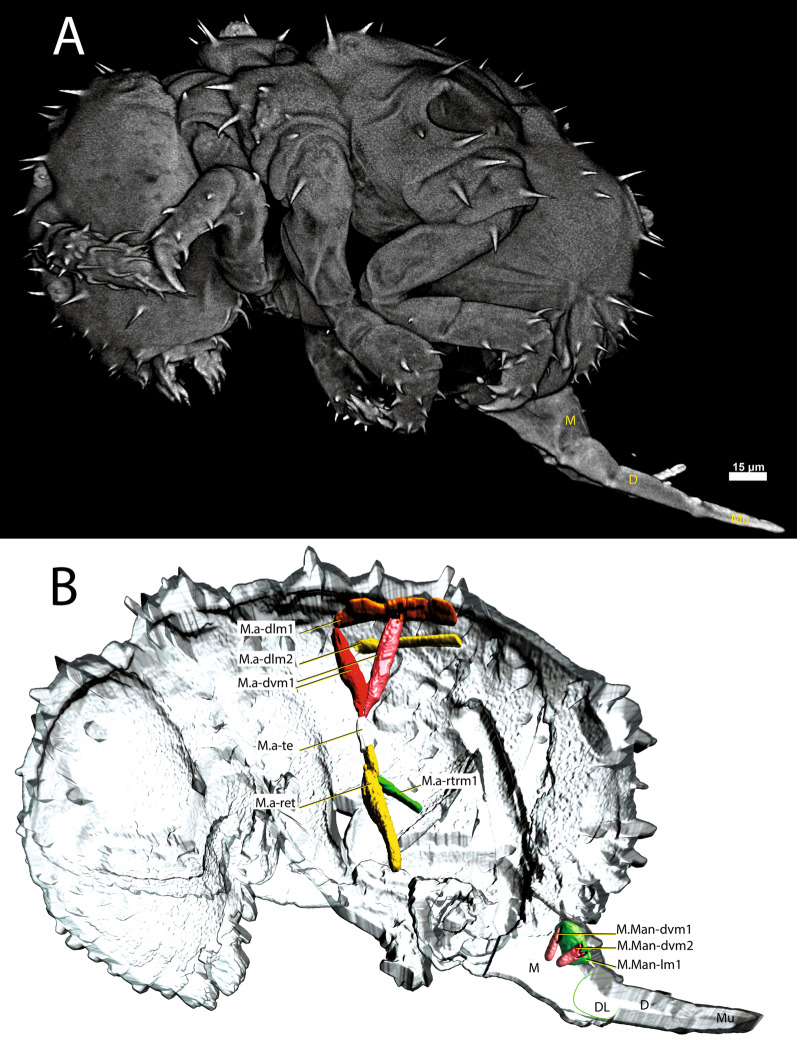
µCT-based morphological reconstruction of the abdominal segments, the jumping apparatus, cuticle and musculature of the retinaculum in *Megalothorax minimus* Willem, 1900 [50]. **A** Side external view of head, thorax and abdomen – extended furca, **B** Cut-away longitudinal view – extended furca. M: manubrium; D: dens; DL: dens lock; Mu: Mucro

**Fig. 11:**
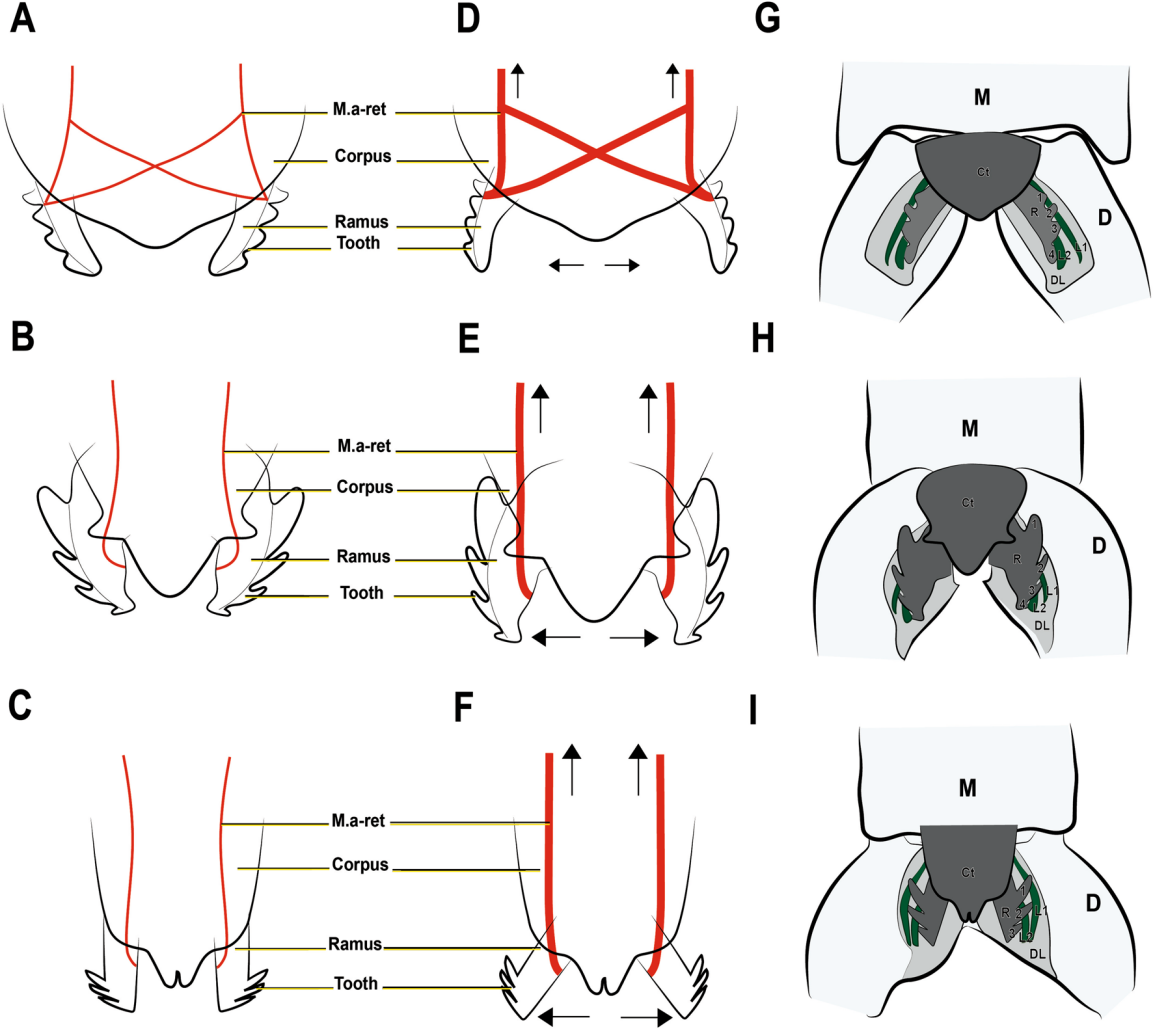
Hypothetical mechanism by which retinaculum muscle sets up the latch between retinaculum and dens lock in the species investigated in this study: **A**, **B** and **C** M.a-ret in relaxed state; **A** *Podura aquatica* Linnaeus, 1758 [26], **B** *Dicyrtomina ornata* (Nicolet, 1842); **C** *Megalothorax minimus* Willem, 1900 [50]. **D**, **E** and **F** M.a-ret contracts to set the retinaculum in the dens lock. **D** *Podura aquatica*; **E** *Dicyrtomina ornata*; **F** *Megalothorax minimus*. **G**, **H**, **I** Latched state, the retinaculum is engaged in the dens lock; **G** *Podura aquatica*; **H** *Dicyrtomina ornata*; **I** *Megalothorax minimus*. M: manubrium; D: dens; DL: dens lock; Ct: corpus tenaculi; R: ramus; L1: Lock 1; L2: Lock 2

M.a-ret inserts ventrally on the medial side of the ramus and runs to the dorsal side where it inserts into a tendon connecting muscles running dorsoventrally (Figs. 10 and 11C). These include M.a-rtrm1, M.a-dvm1 and M.a-dvm2. M.a-rtrm1 connects the M.a-ret muscles with the connecting tendon. Two pairs of dorsal longitudinal muscles, M.a-dlm1 and M.a-dlm2, extend in an anteroposterior direction. No dorsoventrally oriented muscles are present other than those mentioned. Longitudinal muscles are completely absent in the ventral part of the abdomen. In contrast, there are significantly more dorsoventral and transverse muscles in the posterior part of the abdomen than the other studied species. In general, however, the abdominal muscular system in *M. minimus*, especially in the anterior region, has significantly less muscle than in the other species included in this comparative study.

## Discussion

### Lifestyle: adaptation and miniaturization in springtails

*Neanura muscorum* lives mainly in decaying wood and is not able to jump. This correlates with the secondary loss of the furca and retinaculum in this species. Neanuridae is probably the most recently evolved taxon within Poduromorpha [14, 15, 28], which supports the hypothesis that the loss of the furca was secondary. A stunting of the furca occurred independently in several taxa in Collembola [37], including *N. muscorum*. Different representatives exhibit various stages of atrophy, but Pistor [37] concluded that despite the absence of the furca, there is still an increased muscle density in the fourth abdominal segment. The same conclusion was drawn by Bretfeld [8], who explicitly associated the muscles which appear in addition to the segmental musculature with the vestigial furca. One explanation for the extremely complex and strongly interconnected muscular system of the abdomen in *N. muscorum* may be found in this specie’s habitat. Under bark or in dead wood, it might be advantageous for body shape to be able to adapt to the interstitial spaces available. *Podura aquatica*, on the other hand, lives on water surfaces [40] where there is no spatial constriction. The abdominal muscle system in this species, therefore, is less adapted to shape change for the purposes of fitting into fissures and narrow spaces and more adapted to jumping on water surfaces. There are significantly fewer transverse muscles in *P. aquatica* than in *N. muscorum*, but the dorsoventral muscles are more pronounced and thicker. And in *P. aquatica*, dorsal intersegmental longitudinal muscles appear which are not present in *N. muscorum*. Another taxon with distinct segmentation is *Orchesella cincta*. This species lives on the surface of soil and shrubs and is an efficient jumper [32, 41]. According to Schaller [41], animals that inhabit the herbaceous layer like *O. cincta* jump much more frequently than animals that live in the deeper layers of the earth. The morphological adaptations that permit efficient jumping could, then, include an increase in the musculature of the 4th abdominal segment and manubrium.

Symphypleona, the group which includes *Dicyrtomina ornata*, are exceptional jumpers [12]. They mainly live in the grass layer [40] and therefore use their jumping skills much more often than other Collembola [41]. Characteristic in *D. ornata* is the fusion of the thorax with the first four abdominal segments and the absence of ventral longitudinal muscles in the postulated fourth abdominal segment. The same pattern is found in *Megalothorax minimus*. *M. minimus* is the smallest of the species studied and, as mentioned above, has internal and external similarities with *D. ornata*. These include a large number of especially thick dorsoventral muscles associated with the basal plates. In contrast to *D. ornata*, however, *M. minimus* has significantly less muscle in the anterior region of the abdomen, possibly as a result of miniaturization. Panina et al. [35] studied the effects of miniaturization in *Mesaphorura sylvatica* [39], a Tullbergiidae, and found that phenomena such as a reduction in the number of some muscles, a reduction in midgut musculature and the appearance of an unpaired female gonad occur in this taxon. A miniaturization effect that appears in both *M. sylvatica* and *M. minimus* is the reduction mentioned above in the number of muscles in the anterior part of the abdomen. The extreme smallness of *M. minimus* can be explained by its preferred habitat, the euedaphon [41]. Diminutive size can be considered an adaptation to the small interstitial spaces of the soil at this depth, with similar sizes observable in other inhabitants of this soil layer [18]. Euedaphic animals also usually show little to no pigmentation [18]. This, and the absence of occelli [40], also occurs in *M. minimus* as an adaptation to living in the soil.

### Setting up the retinaculum and dens lock as a latch, and the latch release

#### Functioning of the retinaculum

As stated, the retinaculum is a discrete triangular structure with two movable rami. The muscle M.a-ret inserts at the base of the retinaculum ramus and is mainly responsible for the configuration of the retinaculum in the dens lock as a latch. However, the architecture and point of insertion of this muscle in the ramus differ between the taxa studied, which suggests that the mechanical mechanisms may vary between species (Fig. 11A–I). In *P. aquatica*, the M.a-ret attaches laterally to the base of the ramus and its musculature fuses medially (Figs. 4B, 6, 7 and 11), while in *O. cincta* [32], *D. ornata* (Figs. 8, 9 and 11) and *M.minimus* (Figs. 10 and 11) the M.a-ret inserts medially and the muscles are not fused in the middle. In *O. cincta*, it is hypothesized that M.a-ret contraction causes the retinaculum rami to lock themselves in the dens lock to hold the furca when necessary [32]. The same mechanism is hypothesized in this study (Fig. 11A–I) to occur in *D. ornata*, *M. minimus*, and even *P. aquatica*, although the architecture and lateral connection between M.a-ret and the ramus in this last species suggest that the muscles may work in a different way.

### Latching state: insertion of the key (retinaculum) into the lock (dens lock)

The setting up of the rami for the locking into the dens lock must occur simultaneously with the flexion movement of the furca in an anterior direction, allowing physical contact between the two structures (retinaculum and dens lock) to establish the latch. At this point the retinaculum is the key and the dens is the lock, with the teeth on the ramus fitting into the hooked structures (L1 and L2) present in the dens lock. We assume that the distal teeth of the ramus connect with the distal hooks (L1 and L2) on the dens lock as shown in Figs. 11C, F and I. However, exactly where the basal teeth 1 and 2 fit into the dens lock is still an open question.

### Unlatching state: release of the retinaculum from the dens lock

**Fig. 12:**
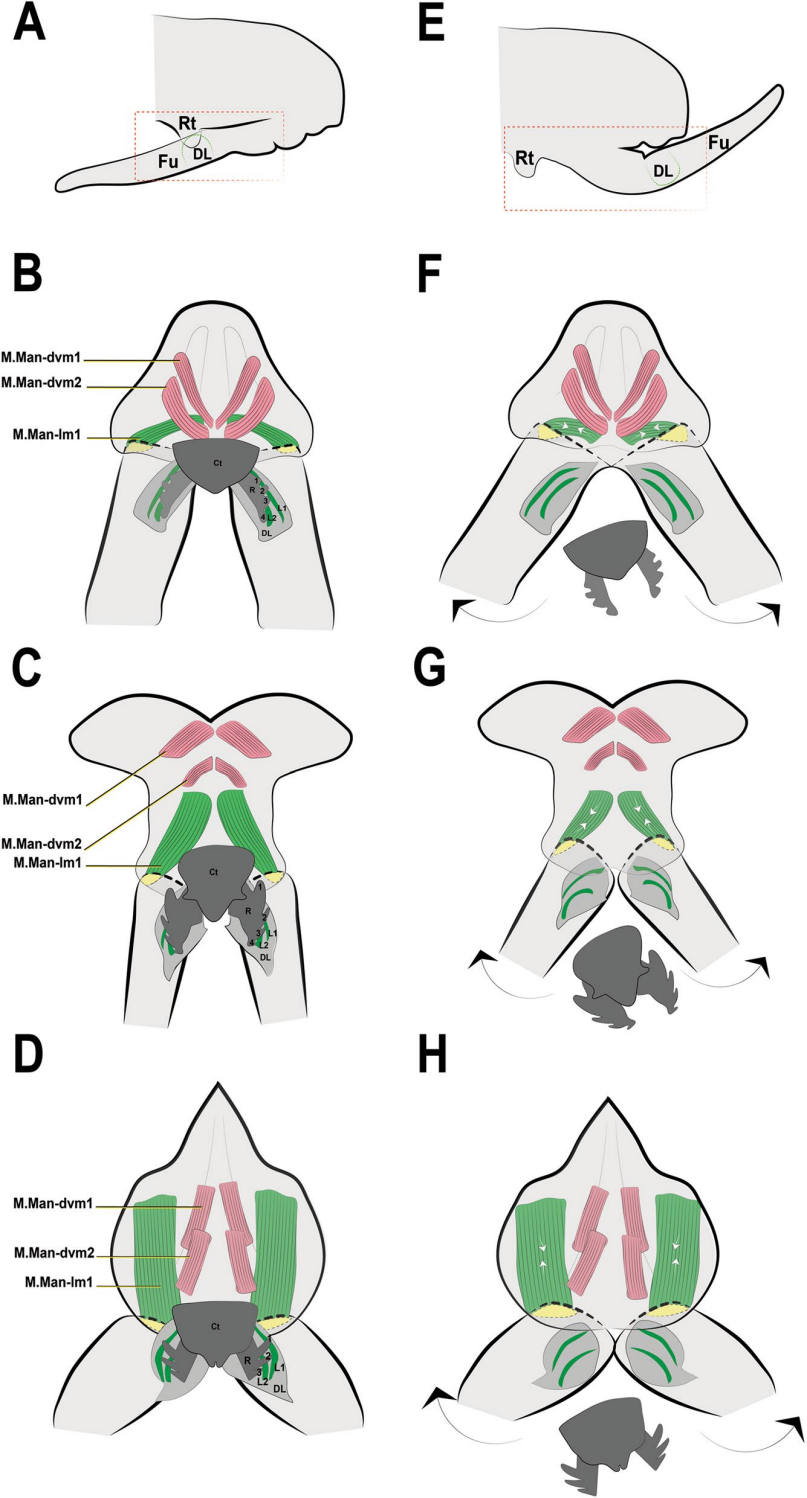
Hypothetical mechanism by which the furca is released from the retinaculum in the different species investigated: **A**–**D** Flexed furca state—Latched phase; **A** Flexed furca and structures of interest; **B** *Podura aquatica* Linnaeus, 1758 [26], **C** *Dicyrtomina ornata* (Nicolet, 1842); **D** *Megalothorax minimus* Willem, 1900 [50]. **E**–**H** Extended furca state—Unlatched phase. **E** Extended furca and structures of interest, **F** *Podura aquatica*; **G** *Dicyrtomina ornata*; **H** *Megalothorax minimus*. Fu: furca; D: dens; DL: dens lock; Rt: retinaculum; L1: Lock 1; L2: Lock 2

It is possible that the force required to release the latch between the retinaculum and the furca is produced in a location independent of the retinaculum, as suggested by Manton [29] and Oliveira [32]. Manton [29] proposed that hemolymph pressure releases the furca from the retinaculum and creates the thrust, and also suggested that the pleural muscles in *Tomocerus longicornis* Müller, 1776 [31] may be directly involved in protecting the flexible basal parts of the body wall against increased hydrostatic pressure and thus strengthening the action of the longitudinal sternal muscles in the vicinity of the retinaculum to release the furca. Oliveira [32] proposed the alternative hypothesis that furca release occurs by muscular force, from the contraction of the manubrial muscle M.Man-lm1 and consequent opening of the dens lock. Such an movement of the dens could be lateral. However, high resolution video images are needed to properly understand this mechanism. This is what is hypothesized to occur in the studied taxa, although the M.Man-lm1 muscle in *P. aquatica* is quite dissimilar in volume to that found in other taxa (Fig. 12A–H). It is also possible that the furca is released from the retinaculum through synchronized body movements prior to jump initiation. Christian [13] described the beginning of the jump in *O. cincta* as starting with a change in the longitudinal axis of the body as the animal inclines the head and trunk towards the ground. Possibly, then touching the ends of the furca (the mucro) to the ground could creates a lateral opening of the dens lock which causes the furca to be released.

#### The role of hemolymph pressure in jumping

Although Oliveira [32] rejected the hypothesis that the hemolymph pressure causes the release of the retinaculum, he considered that it may play a role (alongside the spring mechanism) in increasing the efficiency of jumping in terms of force and distance. Both Manton [29] and Oliveira [32] identified the capacity of the posterior abdominal segments to overlap as being the main mechanism in creating hemolymphatic pressure in segmented springtails (such as Entomobryomorpha). However, this pressure generation mechanism would seem to be absent in Symphypleona and Neelipleona due to the fused segmentation in these taxa. This raises the question of how hemolymph pressure might be created in globular springtails, and whether overlapping segments in Entomobryomorpha really do have anything to do with jumping. No substantial indication was found in this comparative analysis of segmented and globular taxa of the cross-taxa presence of a hemolymph pressure system—for example a channel through which the liquid can flow to create a hemolymphatic pump for use in jumps. We question here the contribution of hemolymphatic pressure to jumping efficiency and credit the success of the jump to the spring mechanism created by the deformation of the basal plates and sclerites [32]. The hypothesis that jumping in Symphypleona is affected purely by a spring mechanism is supported by the fact that dissection of the fifth and sixth abdominal segments does not prevent jumping [7].

#### Ground pattern of the retinaculum and its attachment point at the furca, the dens lock

Cassagnau [11] regarded the ventral sternites of abdominal segments III and IV in *Neanura muscorum* as being vestigial of the retinaculum (Fu_1_) and the furca (Fu_2_ and Fu_3_). Bretfeld [8] reinforced this with an ontogenetic morphological study in *N. muscorum* in which the retinaculum muscle was identified. The structure of the retinaculum has been described as variable, mainly in terms of the number of teeth per ramus, which can vary between 2 and 4 among the groups within Collembola. Due to the homoplasticity of this structure, the number of teeth per ramus can even vary between species of a genus: *Megalothorax minimus* has three teeth, for example, while *M. perspicillum* [43] has only four [42]. However, the shape of the retinaculum is consistent across all groups, consisting of an unpaired body, the corpus tenaculi, and two rami [18, 20]. The cuticular pattern of primary granules is present on the corpus tenaculi and rami in all the species examined for this study, in *O. cincta* [32], and several species of *Megalothorax* spp. [43]. In the specimens we examined, the only area of the retinaculum where the granular cuticular pattern is not found are the teeth, which are located laterally in the ramus (Fig. 2). This is relatively constant across all taxa and can therefore be considered part of the ground pattern of the retinaculum in Collembola.

The attachment point of the retinaculum to the furca, the dens lock, was described for the first time by Oliveira [32] and has received little attention so far (Figs. 3, 11). In all species examined here, the region of the dens lock where the retinaculum attaches consists of two longitudinal hooks and their respective longitudinal furrows. The exact shape of the locks L1 and L2 varies slightly. The main part of the dens lock is covered by the cuticular pattern, except for the apical edge of the locks. These characters are constant across the taxa, which is why they can be considered the ground pattern of the dens lock in Collembola.

#### Framing the interactive mechanism between retinaculum and dens lock as a latch

According to Ilton et al. [24] and Longo et al. [27] a latch is more than just a simple switch or energy release mechanism, as by blocking the movement of the spring, it mediates the point in time, the point in space and the rate at which potential energy is converted into kinetic energy. Our results show that the retinaculum is absent when the furca is absent, which suggests a functional dependency relationship in which the retinaculum's sole function is to hold the furca in preparation for a jump to prevent jumping. Our results and Oliveira's [32] findings in *O.cincta* point to enough features in the retinaculum and dens lock to allow us to frame it as a latching mechanism in a biological system. At the base of each of the rami a pivot point is located, medially or laterally to which the main muscle (M.III-ret) of the retinaculum connects. The functioning of the retinaculum involves two states, latched and unlatched, and the necessary force is generated by physical contact.

## Conclusion

The morphology and muscular pattern of the retinaculum and the dens lock vary between representatives of different habitats. This variation seems to be influenced in particular by segmentation pattern and the presence or absence of a furca. The retinaculum and the dens lock interact in a key-lock relationship. The furca release mechanism and the retinaculum latching mechanism seem to be similar across the taxa examined, functioning through muscle force and not as a result of hemolymph pressure as previously suggested. The M.a-ret is responsible for configuring the rami in the dens lock, while the manubrial M.Man-lm1 releases the dens lock from the retinaculum. This finding leads us to question whether hemolymph pressure really does play a role in jumping at all. We also conclude that the reduction of jumping apparatus in springtails occurred secondarily through a miniaturization process for adaptation to specific habitats. The combination embodied by springtails of a stratified distribution profile and convergent morphological adaptations provides an interesting scenario for future studies on how the jumping apparatus is morphologically expressed in different habitats and how this affects the jumping performance. Here we offer important insights into latching mechanisms, which could be of relevance in the context of the spring mechanism studies, energy storage and amplification in biological systems. We venture that although the latch mechanism in springtails is a biological mechanism, the principle behind it may have the potential to inspire and motivate the creation of biomaterials for use in artificial systems. We reinforce that the use of high speed camera images could provide more insights into latching and unlatching mechanisms in springtails.

## Methods

### Taxa sampling

#### *Neanura muscorum* (Templeton, 1835) [48]

*Neanura muscorum* is about 3.5 mm long. It is ellipsoid in shape and is dorsoventraly oblate. The cuticle is mainly blue in color, and soft and sculpted. As a result of a vestigial furca, *N. muscorum* is unable to jump. The habitus is shown in Fig. 1C. Our specimens were mainly found under the bark of decaying trees and in leaf litter in the soil. The specimens were collected in the following localities: 1) Germany, Wildnisschule Teerofenbrücke, Mecklenburg-Vorpommern, 53° 08′ 09″ N; 14° 20′ 07″ E; x.2020; Entomological aspirator; FGL Oliveira coll. 2) Germany, Rostock, Mecklenburg-Vorpommern, Rostocker Heide, 54° 10′ 52.5″ N 12° 10′ 40.9″ E; v.2021; Entomological aspirator and Photo Bowl; B Rillich coll. The key proposed by Janion et al. [25] was used for identification. Series of analyzed specimens: μCT: 2 specimens, N01 and N02; cLSM: 1 specimen, ST09; SEM: 3 specimens, Neanura01, Neanura02, Neanura03.

#### *Podura aquatica* Linnaeus, 1758 [26]

*Podura aquatica* is about 1–1.2 mm long [40]. Its shape can be described as tubular. The cuticle is mainly pigmented blue-black but can also be reddish [40]. The furca is well developed and reaches the coxa of the second leg when it is flexed [40]. The habitus is shown in Fig. 1D. Our specimens were collected in the following locality: Germany, Warnow in Rostock, Mecklenburg-Vorpommern, on a wet meadow pervaded by canals; 54° 06′ 26.4″ N 12° 08′ 05.4″ E; v.2021; Entomological aspirator and Photo Bowl; FGL Oliveira coll. *P. aquatica* was identified by using the key proposed by Schulz [44]. Series of analyzed specimens: μCT: 2 specimens, P01 and T602; cLSM: 3 specimens, ST01, ST02, ST03; SEM: 2 specimens, Podura01, Podura02.

#### *Megalothorax minimus* Willem, 1900[50]

*Megalothorax minimus* is the smallest of the sampled taxa, ranging in size from 0.2 to 0.3 mm [40]. The thorax and the first four segments of the abdomen are fused as described for *Dicyrtomina ornata* but the 5th and 6th segments are more reduced than in *D. ornata* [5]. The habitus is shown in Fig. 1F. According to Beutel & Pohl [5] *M. minimus* occurs in the soil up to a depth of 6 cm. The specimens were collected in the following locality: Germany, Rostock, Mecklenburg-Vorpommern, Rostocker Heide—forests of *Alnus glutinosa*; 54° 13′ 23.2″ N 12° 12′ 38.2″ E; v.2021; Berlese funnel and Flood; B Rillich coll. Taxon identification was carried out using the key proposed by Bretfeld [9]. Series of analyzed specimens: μCT: 2 specimens, N01 and N02; cLSM: 2 specimens, ST07 and ST10; SEM: 2 specimens: N03, N04 and N05.

#### *Dicyrtomina ornata* (Nicolet, 1842)

*Dicyrtomina ornata* is up to 3 mm in length [40] and has a globular abdomen. Pigmentation is mainly greenish yellow with a very variable dark marking [40]. The thoracic segments and the first four abdominal segments are fused. The 5th and 6th abdominal segments are small and clearly segmented. The habitus is shown in Fig. 1G. This species exhibits very marked jumping behavior as a result of its well-developed furca. The specimens were collected in the following locality: Germany, Rostock, Mecklenburg-Vorpommern, garden of the zoology department Allgemeine und Spezielle Zoologie; 54° 05′ 14″ N 12° 08′ 01″ E; v.2021; Entomological aspirator and White tray; FGL Oliveira & B Rillich coll. Taxon identification was carried out using the key proposed by Bretfeld [9]. Series of analyzed specimens: μCT: 2 specimens, D01 and D02; cLSM: 3 specimens, ST03, ST06, ST08; SEM: 3 specimens, Dicyrtomina01, Dicyrtomina02, Dicyrtomina03.

The sampling methods used to collect soil arthropods (Berlese funnel, Flood, Entomological aspirator and Photo Bowl) were applied as described by Palacios-Vargas & Mejía-Recamier [34].

### Fixation

Two different fixation techniques were used. The individuals destined for visualization by confocal laser scanning microscopy were fixed in PFA (4% in PBS), where they remained for at least one hour. The individuals to be prepared for scanning electron microscopy (SEM) and micro computer tomography (μCT) were fixed in Duboscq-Brasil fluid (alcoholic Bouin’s). This fixative consists of 1 part picric acid, 60 parts formalin (40%), 15 parts glacial acetic acid and 150 parts 80% ethyl alcohol [3].

### Phalloidin staining and confocal laser scanning microscopy

Phalloidin A555 (fluorescent dye 555-I Phalloidin produced by BIOZOL) staining was used to visualize the muscles of the specimens. The specimens were fixed in PFA (4% in PBS) for at least one hour before being washed in PBS (0.1 mol (1x) phosphate-buffered saline) in three steps of 5 min each. After this the specimens were washed in 0.05 NaN_3_ PBS (sodium azide) for 5 min and subsequently washed four times for 25 min each with PBT (PBS + Triton X-100). Finally, the animals were washed in a solution containing 1 μl phalloidin and 1 ml PBT, then incubated for at least 90 min in the dark. The bigger animals were left in the solution for several days to achieve better results. After incubation the specimens were washed three times again in PBS. The first step lasted 3 min and the second and third steps 15 min. The animals were then transferred to PBS 1x + 0.05% NaN_3_. All steps from incubation onwards were performed in the dark because exposure to light can bleach out the phalloidin [19, 32].

The stained specimens were mounted using the “sandwich slide” mounting technique [3]. The animals were placed in a drop of 100% glycerin between two cover glasses of 60 mm × 24 mm each. *Dicyrtomina ornata* had to be mounted in RapiClear 1.47 (produced by SunJin Lab) due to the strength of their pigmentation. In each corner of the cover glasses a piece of modelling clay was placed. Images of the animals prepared in this way were taken with the confocal laser scanning microscope Stellaris 8 made by Leica. Two different excitation wavelengths were used. To make the cuticle visible, its property of autofluorescence [2, 10, 22, 30] was utilized at a wavelength of 405 nm. An excitation wavelength of 555 nm was used to take images of the muscles. In order to be able to scan the stained samples with the CLSM over a period of several days, we stored them in the refrigerator at about 5 °C.

### Scanning electron microscopy (SEM)

#### Critical point-drying

After fixation in Duboscq Brasil, the specimens were washed several times in a graded ethanol series (20%, 30%, 40%, 50%, 60%, 70%, 80%, 90% and 98.8% ethanol), at this stage without staining for tissue contrast. The exposure time for each step was 15 min. The animals were then transferred to the dryer (Leica EMCPD300. LEIT-C-PASTE ((Plano Gmbh)) and metallic needles were used to mount the dried specimens. Small amounts of modeling clay were formed into balls and pressed onto the tip of the needle. The clay acted as an adhesive to which the animals were attached [19, 32].

### Sputter-coating with gold and SEM

The sputter coater EM SCD 004 (BALTEC, Balzers) was used to sputter the surface of the mounted specimens with gold. The needles were inserted into a holder and aligned in such a way that the structures to be photographed were clearly visible from all sides. In some cases, a special sample holder invented by Pohl [38] was used. SEM images were taken using a Merlin VP compact (Zeiss) in the electron microscopic center at the University of Rostock.

### Micro-computer tomography

Specimens fixed in Duboscq-Brasil were washed in a graded ethanol series (20% to 99.8%) as described above. The dried samples were mounted on the tip of a toothpick using liquid glue. As the specimens had not been subjected to staining during the ethanol washing steps, at this point we used a post-washing and critical point dry approach [6]. As a contrast medium, solid iodine (≥ 99.8% p.a.) was placed inside a tube with the mounted samples and left for 1 h. The iodine sublimed and was absorbed by animals to varying degrees depending on the tissue. Then, the specimens were μCT scanned in air using the Zeiss XRadia Versa410 X-ray microscope [19, 32].

### 3D reconstructions

Digital image stacks obtained through μCT or cLSM were processed using the 3D-reconstruction software Amira 2020.2 (FEI Visualization Sciences Group, Zuse Institute Berlin). Data processing mainly involved the segmentation of structures of interest—i.e. the marking of specific structures at regular intervals within the image stack. On the basis of the segmentation, the software is able to create a surface rendering representing a 3D-reconstruction of the morphological structure in question. 3-dimensional surface models provide a better understanding of the orientation of organ systems within an animal and make it possible to reveal and study spatial relationships between coherent organs.

### Software

Figure plates were created using Adobe Illustrator and Adobe Photoshop in Adobe Creative Cloud. cLSM scans were prepared and imaged using Imaris × 64 9.7.2 (developed by Bitplane).

### Naming the muscles

To compare the muscle systems of different Collembola it was necessary to describe and name muscles. The nomenclature proposed by Bretfeld [8] was used as an orientation aid.

The names used here consist of three parts. Example: M.IIIa-ldvm2. The prefix provides information about the kind of structure in question. M stands for a muscle in the example given. Names of tendons would start with a T, endosclerites with an E and so on. The abbreviation used to denote the type of structure is always a capital letter separated from a Roman numeral by a dot. The Roman numeral plus the connected lowercase letter are the stem of the name. The Roman numeral shows the segment in which the anterior attachment point of the structure in question is located. In the example given, it is the 3rd abdominal segment. The lowercase letter directly connected to the Roman numeral indicates whether the segment is cephalic (c), thoracic (t) or abdominal (a). When it is not possible to reliably determine the segment to which the muscles connect (in the case of globular springtails), the muscle is named according to the structure to which it connects, such as the retinaculum, for example. In this case, the letter (r) would be included at the beginning of the suffix. The stem is separated from the suffix by a hyphen. The suffix consists of an abbreviation and an Arabic number. The different abbreviations represent different orientations of muscles in the body.

There are four major muscle orientations: longitudinal, dorsoventral, transversal and intersegmental. Some muscle names contain an addendum specifying their exact location (e.g. lateral, ventral, etc.). Longitudinal muscles are defined as muscles running approximately parallel to the longitudinal axis of the animal. Transversal muscles are muscles with an orthogonal orientation to the longitudinal axis. Dorsoventral muscles are a special kind of transversal muscle which are orientated parallel to the sagittal plane and connect the dorsal and ventral sides of the body. The anterior attachment point of intersegmental muscles is located in a different segment than the posterior one. These muscles can be orientated dorsoventrally or longitudinally. In addition to the abbreviation denoting orientation, the suffix contains an Arabic number. In most cases there will be more than one muscle with the same orientation in a segment so it is necessary to distinguish them by counting them and allocating each a number. The way muscles are counted follows different rules for different types. Longitudinal muscles are counted by their anterior attachment point from central to peripheral. Transversal muscles are counted by their ventral attachment point from anterior to posterior. In the special case of transversal muscles orientated in the horizontal plane, the medial attachment point is counted from anterior to posterior. Dorsoventral and intersegmental muscles are counted as described for transversal muscles or, in the case of longitudinally orientated intersegmental muscles, counted as described for longitudinal muscles.

Muscles that divide are named according to their main orientation. To identify individual muscle branches, a dot and another number are added after the first Arabic numeral. Muscle branches are named according to the same principles that apply to the main muscle. In many cases, one of the two attachment points will be described as a muscle center. In this study, a muscle center is defined as a structure to which more than two muscles attach and which is not the inner side of the cuticle. A muscle center could be a tendinous knot or an endocuticular structure, for instance.

## Supplementary Information

**Additional file 1.** Three-dimensional (3D) model µCT-based of morphological reconstruction of the third abdominal segment, cuticle and musculature in *Neanura muscorum* (Templeton, 1835) – transverse view.

**Additional file 2.** Three-dimensional (3D) model µCT-based of morphological reconstruction of the third abdominal segment, cuticle and musculature in *Podura aquatica* Linnaeus, 1758 – transverse view.

**Additional file 3.** Three-dimensional (3D) model µCT-based morphological reconstruction focused on the abdomen, cuticle and musculature related to the retinaculum in *Dicyrtomina ornata* (Nicolet, 1842) – transverse view.

**Additional file 4.** Three-dimensional (3D) model µCT-based morphological reconstruction focused on the abdomen, cuticle and musculature related to the retinaculum in *Megalothorax minimus* Willem, 1900 – cut-away longitudinal view.

## Data Availability

The datasets used and/or analysed during the current study are available from the corresponding author on reasonable request.

## Abbreviations

oc: Eyes
an: Antenna
vt: Ventral tube
M: Manubrium
Ct: Corpus tenaculi
D: Dens
Rt: Retinaculum
Fu: Furca
Mu: Mucro
PA: Anterior part
PP: Posterior part
FMS: Furcular manubrial sclerite
BF: Basal furrow
L1: Lock 1
L2: Lock 2
F1: Furrow 1
F2: Furrow 2
dl: Dens lock
r: Ramus
dlm: Dorsal longitudinal muscles
vlm: Ventral longitudinal muscles
llm: Lateral longitudinal muscles
lm: Longitudinal muscles
dvm: Dorsoventral muscles
ldvm: Lateral dorsoventral muscles
isvlm: Intersegmental ventral longitudinal muscles
ldvm: Lateral dorsoventral muscles
trm: Transversal muscles
istrm: Intersegmental transversal muscle
ret: Retinaculum muscle
PBS: Phosphate buffered saline
PFA: Paraformaldehyde
SEM: Scanning electron microscopy
µCT: Micro-computed tomography
cLSM: Confocal laser scanning microscopy
