# Supplementary figures and images for: On latches in biological systems: a comparative morphological and functional study of the retinaculum and the dens lock in Collembola
Source: Front Zool. 2023 May 9;20:16. doi: 10.1186/s12983-023-00491-2 (PMC10169344; doi:10.1186/s12983-023-00491-2)

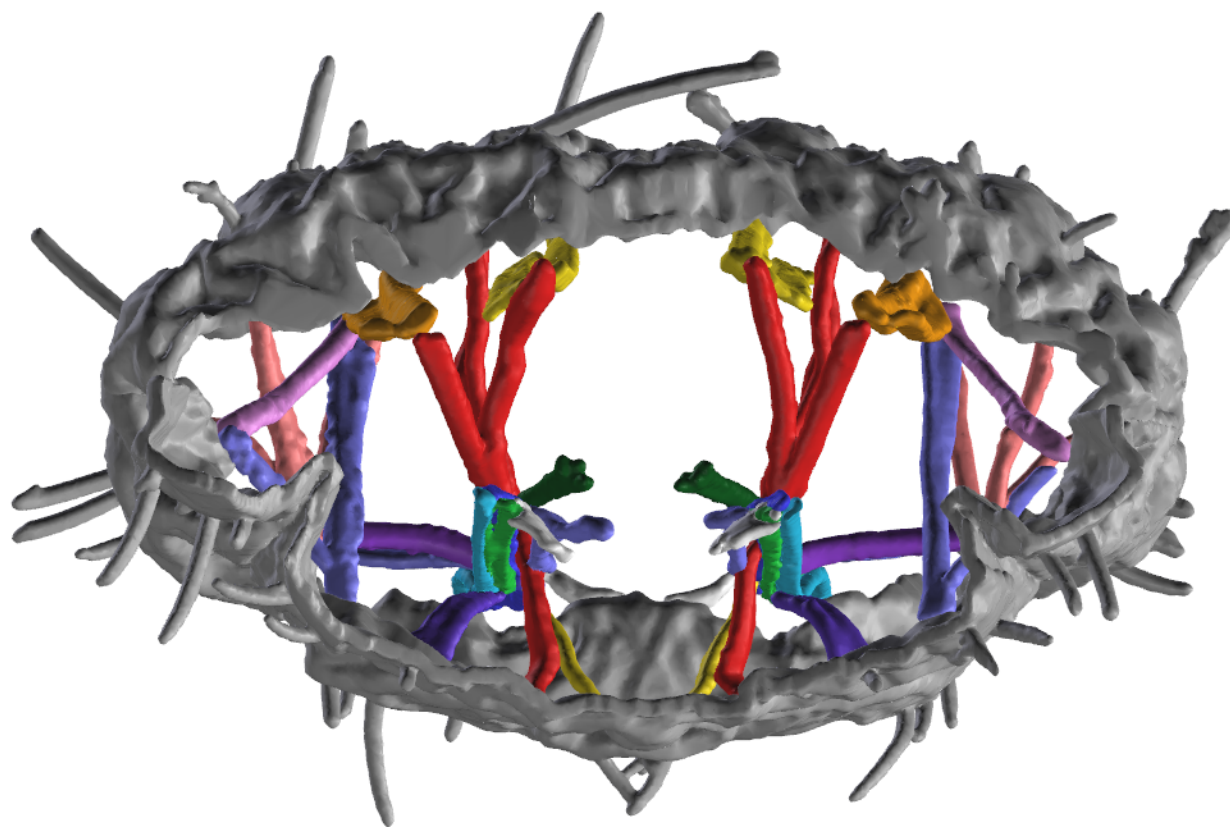

Supplement: Supplementary file 1 — Additional file 1. Three-dimensional (3D) model µCT-based of morphological reconstruction of the third abdominal segment, cuticle and musculature in Neanura muscorum (Templeton, 1835) – transverse view. [file 12983_2023_491_MOESM1_ESM.pdf]

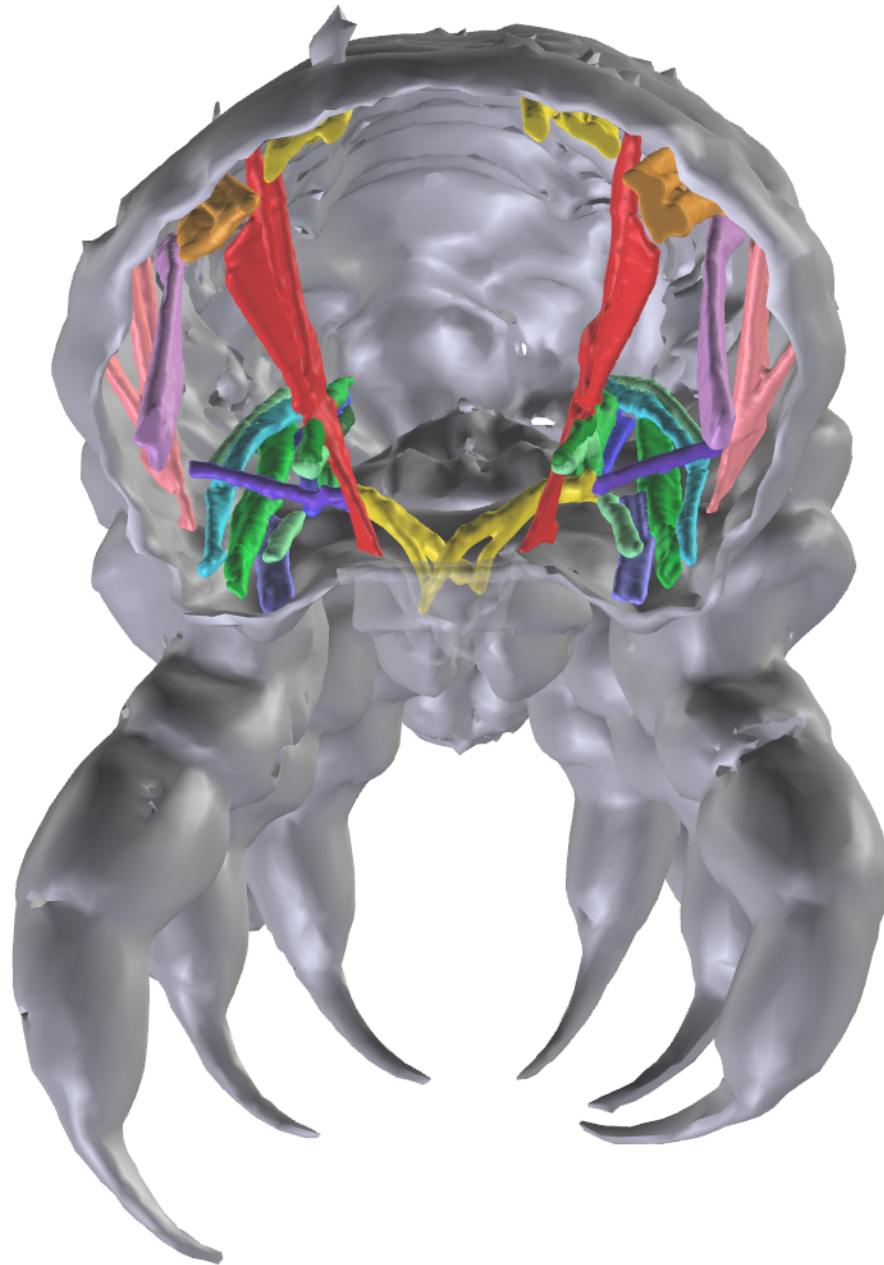

Supplement: Supplementary file 2 — Additional file 2. Three-dimensional (3D) model µCT-based of morphological reconstruction of the third abdominal segment, cuticle and musculature in Podura aquatica Linnaeus, 1758 – transverse view. [file 12983_2023_491_MOESM2_ESM.pdf]

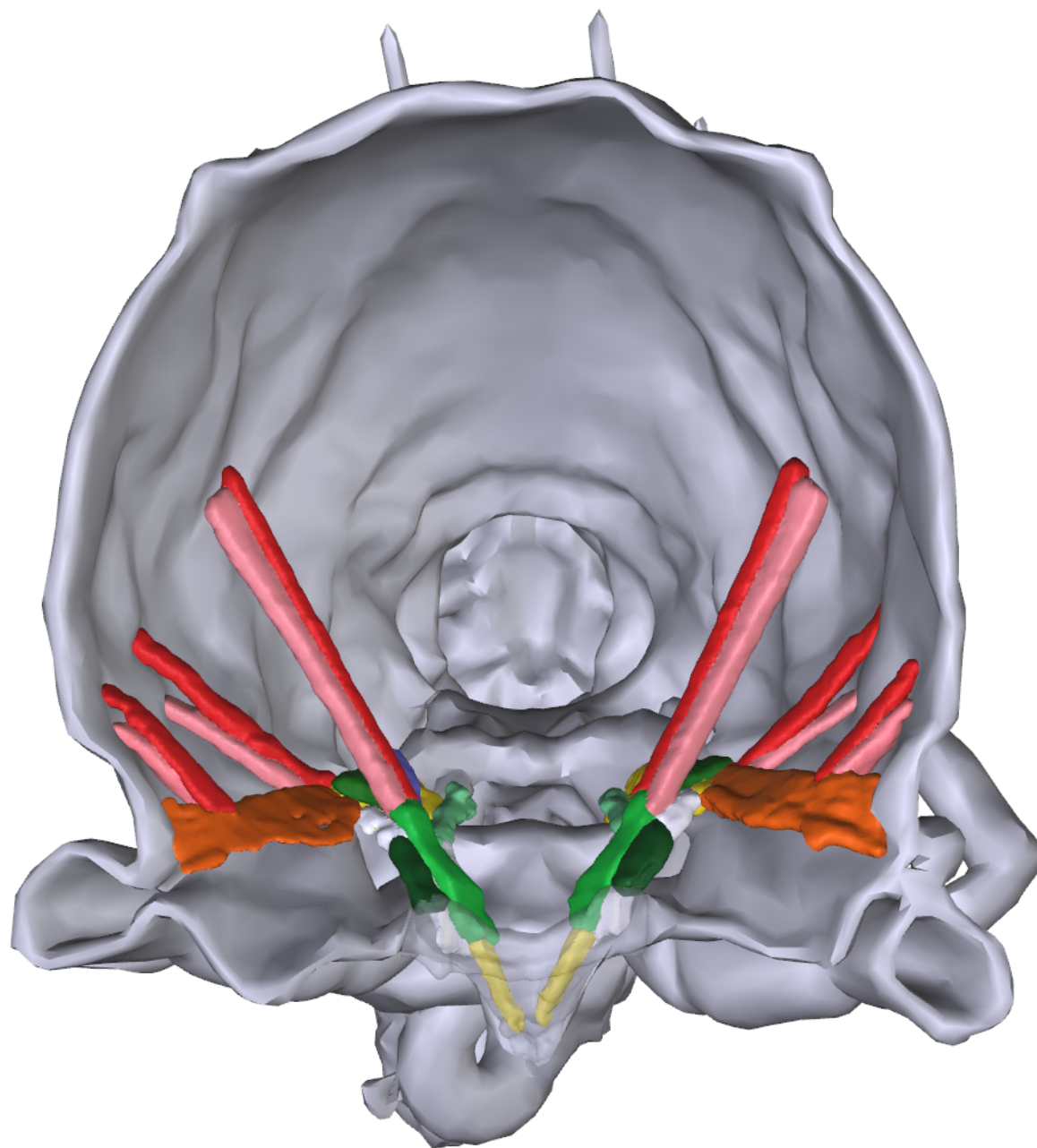

Supplement: Supplementary file 3 — Additional file 3. Three-dimensional (3D) model µCT-based morphological reconstruction focused on the abdomen, cuticle and musculature related to the retinaculum in Dicyrtomina ornata (Nicolet, 1842) – transverse view. [file 12983_2023_491_MOESM3_ESM.pdf]

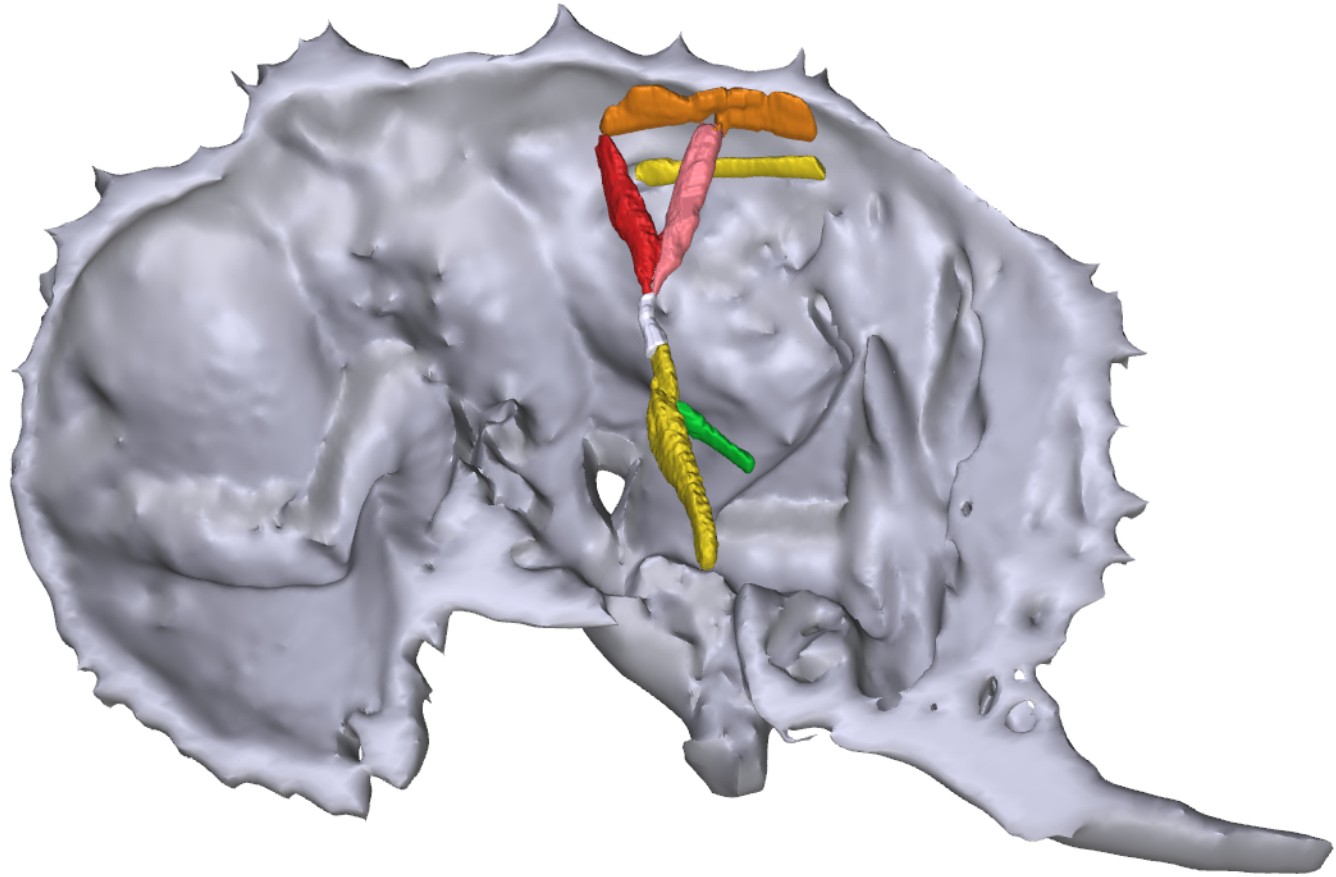

Supplement: Supplementary file 4 — Additional file 4. Three-dimensional (3D) model µCT-based morphological reconstruction focused on the abdomen, cuticle and musculature related to the retinaculum in Megalothorax minimus Willem, 1900 – cut-away longitudinal view. [file 12983_2023_491_MOESM4_ESM.pdf]
